# Cerebrospinal fluid and blood neurofilament light chain levels in amyotrophic lateral sclerosis and frontotemporal degeneration: A meta‐analysis

**DOI:** 10.1111/ene.16371

**Published:** 2024-06-27

**Authors:** Federico Verde, Sara Licaj, Davide Soranna, Nicola Ticozzi, Vincenzo Silani, Antonella Zambon

**Affiliations:** ^1^ Department of Neurology and Laboratory of Neuroscience IRCCS Istituto Auxologico Italiano Milan Italy; ^2^ Department of Pathophysiology and Transplantation, Dino Ferrari Center Università degli Studi di Milano Milan Italy; ^3^ Department of Statistics and Quantitative Methods University of Milano‐Bicocca Milan Italy; ^4^ Biostatistics Unit IRCCS Istituto Auxologico Italiano Milan Italy

**Keywords:** amyotrophic lateral sclerosis (ALS), biomarkers, cerebrospinal fluid, frontotemporal dementia (FTD), neurofilament light chain (NFL)

## Abstract

**Background and purpose:**

Neurofilament light chain (NFL) has been shown to be increased in amyotrophic lateral sclerosis (ALS) and, to a lesser extent, in frontotemporal dementia (FTD). A meta‐analysis of NFL in ALS and FTD was performed.

**Methods:**

Available studies comparing cerebrospinal fluid and blood NFL levels in ALS versus neurologically healthy controls (NHCs), other neurological diseases (ONDs) and ALS mimics, as well as in FTD and related entities (behavioural variant of FTD and frontotemporal lobar degeneration syndromes) versus NHCs, ONDs and other dementias were evaluated.

**Results:**

In ALS, both cerebrospinal fluid and blood levels of NFL were higher compared to other categories. In FTD, behavioural variant of FTD and frontotemporal lobar degeneration syndromes, NFL levels were consistently higher compared to NHCs; however, several comparisons with ONDs and other dementias did not demonstrate significant differences.

**Discussion:**

Amyotrophic lateral sclerosis is characterized by higher NFL levels compared to most other conditions. In contrast, NFL is not as good at discriminating FTD from other dementias.

## INTRODUCTION

Amyotrophic lateral sclerosis (ALS) is a neurodegenerative disease affecting upper motor neurons of the motor cortex and lower motor neurons of the brainstem and spinal cord. It causes progressive paralysis of voluntary muscles leading to death after a median time of 3 years from symptom onset. Approximately 10% of cases are familial, caused by genetic mutations in one of >30 genes, usually with autosomal dominant inheritance. The diagnosis is mainly clinical and is supported by electromyography [1].

Frontotemporal dementia (FTD) is the third most common form of degenerative dementia after Alzheimer's disease (AD) and dementia with Lewy bodies. It has a strong genetic component, with up to one‐third of cases having a positive family history, most commonly due to dominantly inherited mutations in one of three main genes (*MAPT*, *GRN* or *C9orf72*). FTD is subdivided into three clinical entities: the behavioural variant (bvFTD) and two forms of primary progressive aphasia (PPA), that is, the non‐fluent variant (nfvPPA) and the semantic variant (svPPA) [2]. The three FTD subtypes belong to the wider category of the so‐called frontotemporal lobar degeneration (FTLD) syndromes (FTLDSs), that is, the clinical diagnoses associated with the neuropathological entity FTLD. This group also comprises the two atypical parkinsonian syndromes progressive supranuclear palsy and corticobasal syndrome. FTLD features degeneration of the frontal and temporal lobes and, in turn, is subdivided into two main pathological subtypes, characterized by intracellular deposits of the proteins TDP‐43 (FTLD‐TDP) and tau (FTLD‐tau), respectively [3]. Intracellular TDP‐43 inclusions are also the neuropathological hallmark of ALS. Therefore, the term TDP‐43 proteinopathies has been coined to designate both ALS and FTLD‐TDP [4]. Indeed, the two entities also share genetic underpinnings and can be associated clinically to a variable extent [5, 6].

For both ALS and FTD, research on neurochemical biomarkers is highly active. Biomarkers have the potential to aid diagnosis, especially early in the disease course, to help define prognosis and, in the context of therapeutic experimentations, to screen and stratify patients, to demonstrate target engagement and to measure treatment effects [7, 8]. Amongst the most extensively studied biomarkers for ALS and FTD are neurofilaments, which are abundant structural components of the cytoskeletal scaffold of axons of the central and peripheral nervous system [9]. Amongst the three neurofilament subunits (light, intermediate and heavy chains), neurofilament light chain (NFL) is the most studied biomarker. In the case of neuroaxonal damage of any kind, NFL is released from the axons into the extracellular space of the brain or spinal cord, so that its levels in the cerebrospinal fluid (CSF) rise. From here, NFL then reaches the blood, albeit at much lower concentrations. Thanks to technological advances of the last 10 years, levels of NFL in blood (plasma or serum) can now be quantified, turning out to be increased in the same conditions of neuroaxonal injury as CSF levels. This offers great advantages compared to CSF in terms of ease of sampling, reduced invasiveness, costs and repeatability [10]. As a marker of axonal damage, NFL is, in general, rather unspecific. However, in ALS, its CSF and blood levels are particularly increased due to the intense degeneration of the long axons of motor neurons, so that they are reported to differentiate ALS from most other neurodegenerative diseases. Moreover, in ALS, NFL levels are amongst the most valuable prognostic predictors, as they correlate with disease progression rate and are negatively associated with survival [11, 12, 13]. Importantly, plasma NFL has been accepted as a pharmacodynamic biomarker in randomized controlled trials evaluating experimental ALS therapeutics [14]. On the other hand, in FTD, NFL levels are less markedly increased; however, in addition to being elevated compared to neurologically healthy controls, they tend to be higher than in AD and, relevantly for differential diagnosis, in primary psychiatric disorders [15, 16]. Furthermore, they are negatively associated with measures of cognition and brain volume [17]. A large single‐centre study also provided evidence that CSF NFL levels are significantly higher in probable (clinically defined) or definite (genetically or neuropathologically defined) FTLD‐TDP compared to probable or definite FTLD‐tau, with an area under the receiver operating characteristic curve of 0.861 and with sensitivity and specificity of 80.0% and 81.0%, respectively [18]. Importantly, in healthy individuals, NFL levels increase with increasing age, possibly as a consequence of subclinical age‐related neurodegeneration [19].

The role of NFL as a diagnostic biomarker for ALS and FTD has been previously addressed by reviews and meta‐analyses [10, 20, 21]. However, this field is rapidly expanding and a further body of evidence has been produced in the last few years. Moreover, to our knowledge, the role of NFL has not been evaluated to date in the context of a single meta‐analysis focusing on both ALS and FTD, and previous appraisals have not stratified the assessed investigations based on relevant quality parameters such as age balance between case and control cohorts. Therefore, a meta‐analysis of available evidence on CSF and blood levels of NFL was performed in patients with ALS and with FTD and related entities (bvFTD and FTLDSs) compared to healthy controls and patients with other neurological diseases.

## METHODS

### Search strategy and selection criteria

A search on PubMed and Web of Science was performed up to 24 February 2022, using the following search strategy: (i) for ALS ([amyotrophic lateral sclerosis OR ALS] AND [neurofilament light chain OR NFL]); (ii) for bvFTD/FTD/FTLDSs ([frontotemporal dementia OR FTD OR frontotemporal lobar degeneration OR FTLD] AND [neurofilament light chain OR NFL]). The references of previous meta‐analyses, systematic reviews and other relevant publications were hand‐checked to assess the completeness of the studies retrieved by the literature search. Studies were included in the meta‐analysis if (1) cases were clearly identified as ALS, bvFTD, FTD or FTLDSs, or there was sufficient information to classify them; (2) one or more control groups were included; (3) control groups could be identified as neurologically healthy controls (NHCs), ALS mimics (AMs) (i.e., conditions clinically resembling ALS, for studies including ALS), other dementias (ODs) (for studies including FTD and related entities) or other neurological diseases (ONDs); (4) mean, or median, and standard deviation, or interquartile range, of NFL levels in CSF or blood (plasma or serum) were reported. In the case of duplicated data, only the most recent publication was selected. Two authors (SL and FV) independently assessed the eligibility of each paper; disagreements between readers were solved by discussion.

From the included papers, the following information was extracted: first author and year of publication, geographical area, types of cases, types of controls, sample size, mean age of the study participants, proportion of male participants, biological fluids analysed (CSF or blood, i.e., plasma or serum).

### Statistical analysis

Neurofilament light chain levels reported as median and interquartile range were transformed to mean and standard deviation [22] before applying the meta‐analytic procedure. The mean difference between cases and controls was standardized following Cohen's approach (standardized mean difference, SMD) [23]. For each comparison, the SMDs were pooled weighting each estimate by its inverse of variance. A random effect model was considered to take into account the between‐study variability (*𝜏* ^2^) using the Sidik–Jonkman estimator. Moreover, the Hartung–Knapp–Sidik–Jonkman method was applied to accurately estimate the 95% confidence interval of the pooled SMD [23]. An important aspect which was taken into account in each paper was the comparability between cases and controls for age obtained by design (matching). Therefore, in the forest plots three pooled SMD estimates are reported: the first regarding studies with different mean ages between groups (stratum A), the second regarding studies with similar mean ages (stratum B) and the third considering all studies (stratum Overall). Each pooled SMD estimate was calculated only when at least three original SMDs were available. The heterogeneity between studies was evaluated by means of the *I* ^2^ of Higgins and Thompson [24]. In the case of marked heterogeneity (*I* ^2^ more than 75% [25]), when at least 10 studies were available, a meta‐regression model was fitted to analyse the sources of heterogeneity [26]. The covariates used to explain the heterogeneity were mean age and proportion of males amongst cases, number of cases and controls, comparability between cases and controls for age, and publication year. The robustness of the findings was evaluated by applying an influence analysis obtained by omitting one study at a time. Potential publication bias was investigated using a funnel plot as a visual inspection of the bias, and, when at least 10 studies were provided, also Egger's regression test [27]. Analyses were performed using R, version 4.2.3.

## RESULTS

Regarding ALS, the initial search provided 529 records, which were reduced to 208 after removing duplicates. After excluding 107 non‐relevant articles, the 101 remaining articles were assessed for eligibility. This resulted in a final number of 56 studies included in the meta‐analysis (Figure S1.). As to FTD and related entities (bvFTD and FTLDSs), the initial search identified 390 records. After the removal of 249 duplicates, 141 articles were screened, of which 38 were excluded as non‐relevant. The remaining 103 papers were assessed for eligibility, resulting in a final number of 62 studies included in the meta‐analysis (Figure S2).

In the main text the meta‐analytic pooled estimates (Figures 1, 2, 3, 4) for each comparison of interest are reported, whilst the corresponding forest plots are reported in Figures S3–S14. First, CSF NFL levels in ALS were compared to NHCs, AMs and ONDs. CSF NFL levels were higher in ALS compared to the three other groups, and this applied to studies of stratum A, to those of stratum B, and when considering all studies together (stratum Overall). High *I* ^2^ levels were reported for the comparisons with NHCs and ONDs, whereas *I* ^2^ levels were lower for the comparison with AMs (Figure 1).

**FIGURE 1:**
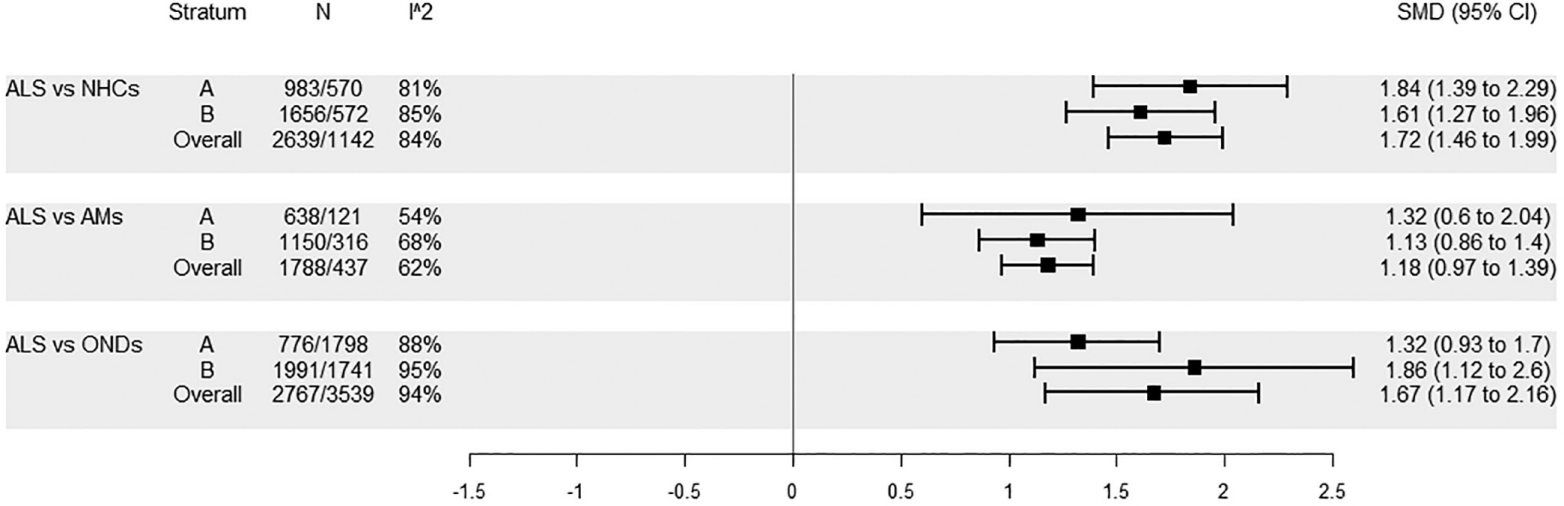
Comparisons pertaining to group ALS, CSF: A, no similar age between cases and controls; B, similar age between cases and controls.

**FIGURE 2:**
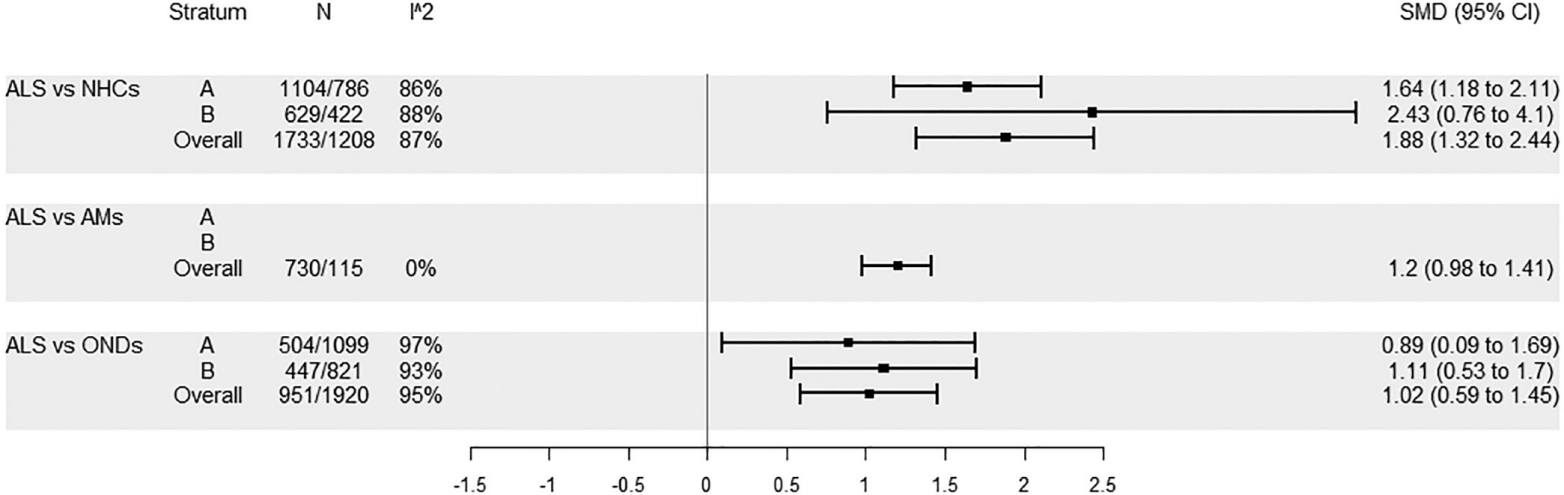
Comparisons pertaining to group ALS, blood: A, no similar age between cases and controls; B, similar age between cases and controls.

As regards blood NFL levels, our findings were similar, with ALS showing higher levels compared to NHCs, AMs and ONDs for all strata. Also in this case *I* ^2^ levels were high for the comparisons with NHCs and ONDs, whereas *I* ^2^ was 0 in the comparison with AMs. Because of the low number of studies, the pooled estimates of strata A and B are not reported (Figure 2).

**FIGURE 3:**
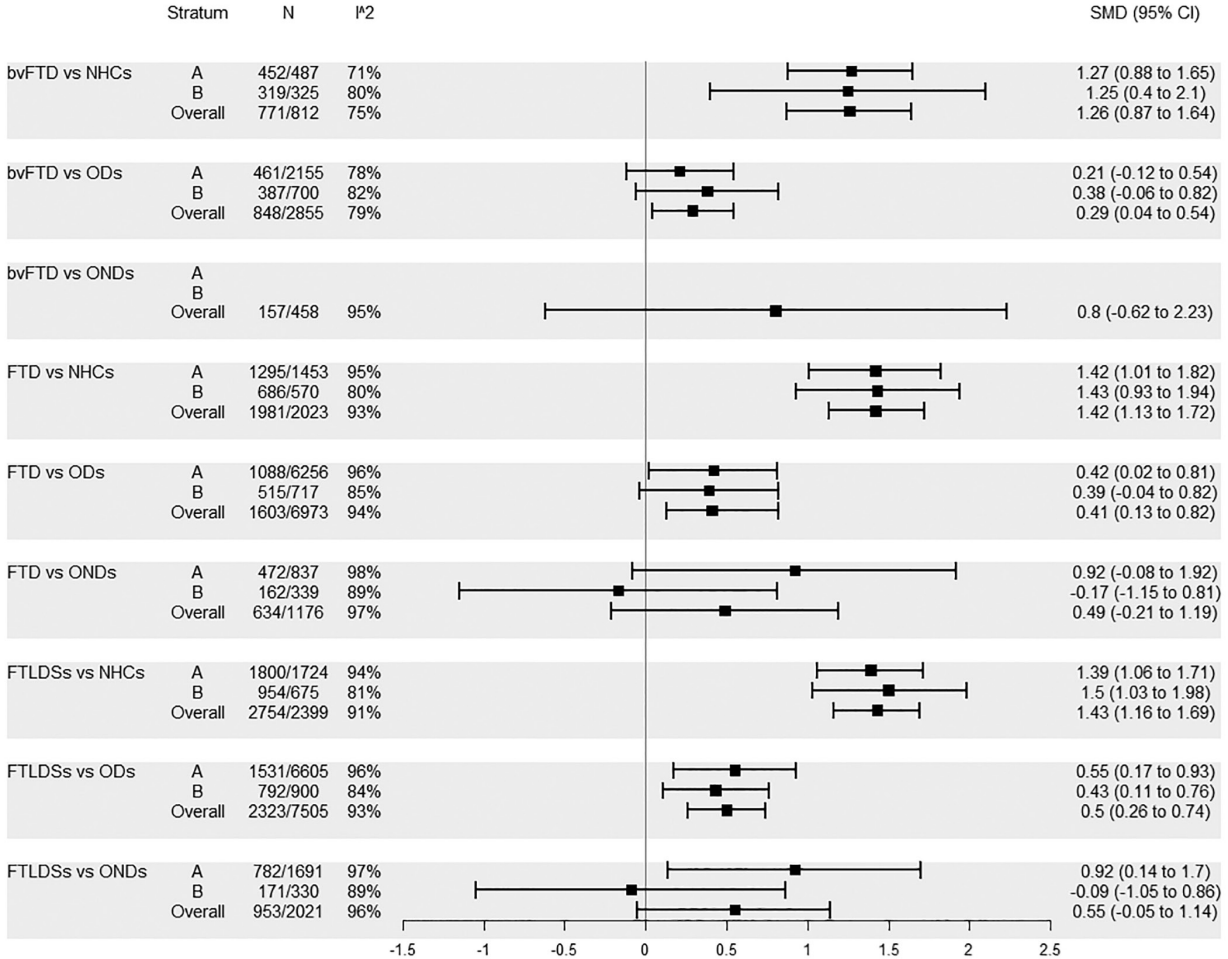
Comparisons pertaining to groups bvFTD, FTD and FTLDSs, CSF: A, no similar age between cases and controls; B, similar age between cases and controls.

Then NFL levels in FTD and related entities were analysed, again starting from the CSF. Here, high *I* ^2^ levels were found in almost all comparisons. bvFTD was characterized by higher CSF NFL levels compared to NHCs in all strata. On the other hand, in the comparison with ODs, bvFTD had significantly higher CSF NFL levels in stratum Overall, but not in stratum A and stratum B. The comparison between bvFTD and ONDs was performed only in stratum Overall, with no significant difference in CSF NFL levels. The comparison between FTD and NHCs was similar to that reported for bvFTD. Differently from what was observed for bvFTD, for FTD not only stratum Overall but also stratum A showed significantly higher CSF NFL levels compared to ODs. As already reported for bvFTD, no significant differences in CSF NFL levels compared to ONDs were found for FTD. On the other hand, FTLDSs were characterized by higher CSF NFL levels compared to both NHCs and ODs in all strata, whereas in the comparison with ONDs this held true only for stratum A (Figure 3).

**FIGURE 4:**
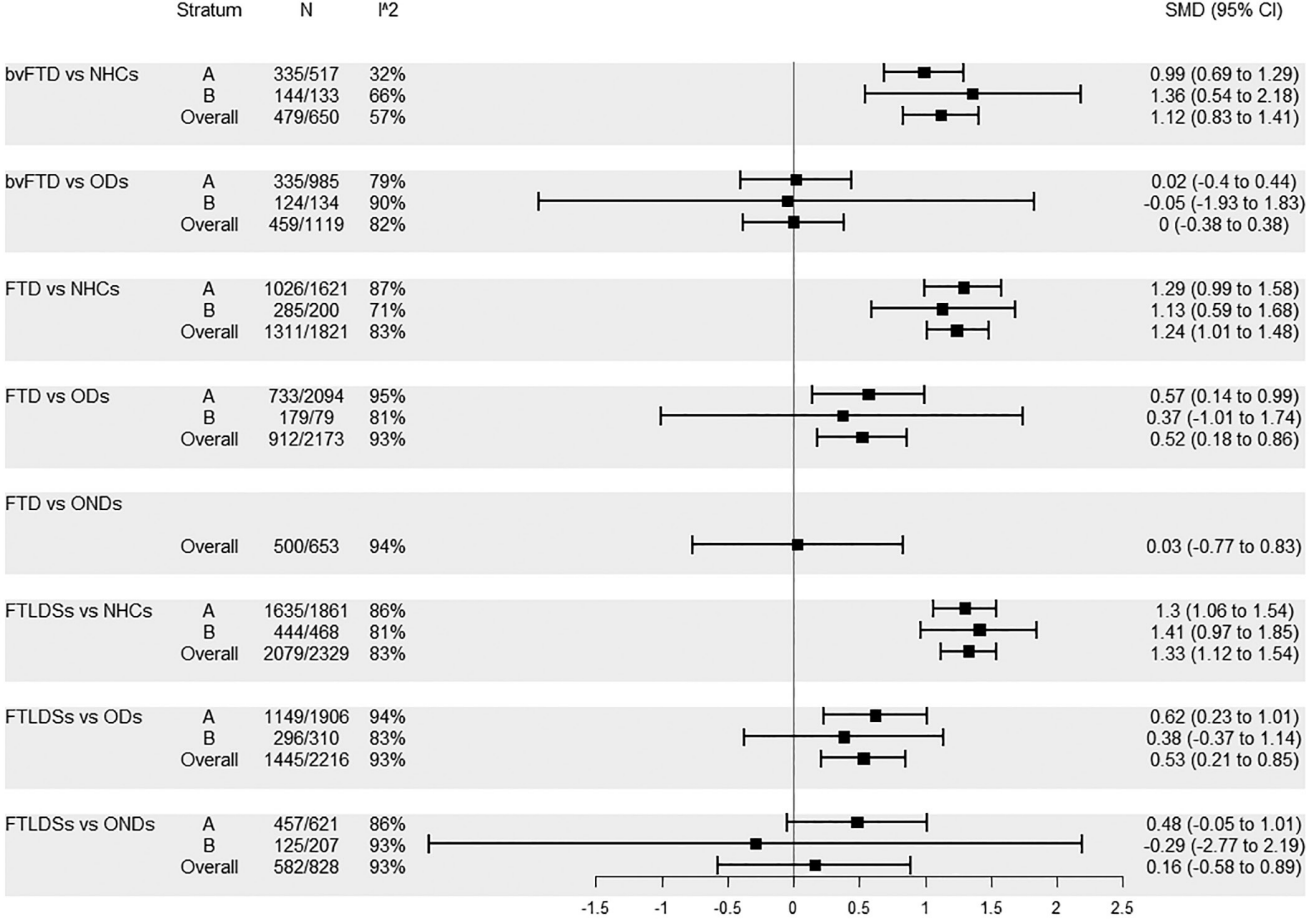
Comparisons pertaining to groups bvFTD, FTD and FTLDSs, blood: A, no similar age between cases and controls; B, similar age between cases and controls.

Finally, the comparisons regarding blood NFL in FTD and related entities were also characterized by high *I* ^2^ values, with the notable exception of that between bvFTD and NHCs. bvFTD was characterized by significantly higher blood NFL levels compared to NHCs (in all strata) but not compared to ODs or ONDs (for the latter comparison the pooled estimate was limited to stratum Overall). For FTD and FTLDSs similar results were observed with regard to the comparisons with NHCs and ONDs, whilst, in the comparison with ODs, the pooled SMD was statistically higher for all strata except for stratum B (Figure 4).

The results of influence analysis, publication bias analysis and meta‐regression are reported in the Supporting Information. Influence analysis (Figures S15–S26) showed that all findings were robust when omitting one article at a time. Evidence of publication bias was found only for the comparisons ALS versus NHCs (for both CSF and blood, Egger's test, *p* = 0.0077 and *p* = 0.0139, respectively), ALS versus ONDs (for CSF, *p* = 0.0054), bvFTD versus NHCs (for CSF, *p* = 0.0102) and FTLDSs versus ODs (for blood, *p* = 0.0282) (Figure S27). Finally, meta‐regression failed to identify important covariates explaining the heterogeneity between study‐specific estimates. Indeed, only in four comparisons (three regarding CSF and one regarding blood) was at least one variable statistically significant: number of cases/controls and proportion of males amongst cases for FTD, bvFTD and FTLDSs versus NHCs regarding CSF, and number of controls for FTD versus ODs regarding blood (Table S1.).

## DISCUSSION

In our meta‐analysis the differences in CSF and blood levels of NFL were examined between the two neurodegenerative diseases ALS and FTD (and related entities) and other diagnostic groups. Regarding ALS, it was found that both CSF and blood levels of NFL are consistently and significantly higher compared to NHCs, ONDs and, importantly, AMs. For FTD and related entities, the results were less straightforward. In FTD, bvFTD and FTLDSs, CSF as well as blood levels of NFL were consistently higher compared to NHCs; however, the comparisons with ODs and ONDs failed in many cases to demonstrate statistically significant differences.

Features of certain individual studies might have contributed to non‐significant findings in the comparisons of CSF or blood levels of NFL between FTD (and related entities) and other conditions. For instance, the lack of significant differences in CSF NFL levels between FTD and ODs in stratum B studies might have been determined by three studies including patients with Creutzfeldt–Jakob disease (CJD), which is known to be associated with massive increases in CSF and blood NFL [28, 29, 30]. The same may apply to the absence of significant differences of CSF NFL levels in bvFTD compared to ODs in studies of both strata considered separately [28, 29, 31, 32], the other main reason here (not shared with the previous issue regarding FTD in general) being the inclusion of other FTD categories featuring slightly higher NFL levels than bvFTD, namely nfvPPA and especially svPPA [33, 34], in the group of ODs. Similar considerations can be made regarding the finding of non‐significant differences in blood NFL levels between bvFTD and ODs, with the study of Zerr et al. [35] being focused on CJD and other cohorts including patients with FTD variants other than the behavioural one, in particular PPA (nfvPPA and svPPA, the latter being characterized by higher NFL levels) [36, 37, 38, 39]. On the other hand, the lack of significant differences in blood NFL levels between FTD and ODs in stratum B studies can be mainly attributed to the investigation of Matías‐Guiu et al., where the median plasma NFL value of patients with AD was non‐significantly higher than that of patients with FTD [37]. This also partly explains the absence of significant differences in blood NFL levels between FTLDSs and ODs in stratum B studies, another reason being the inclusion of CJD cases amongst patients with ODs in the investigation of Halbgebauer et al. [30]. The abovementioned findings contribute to the lack of significant differences in both CSF and blood NFL levels in FTD, bvFTD and FTLDSs versus ONDs, as the latter also include ODs. However, another major factor preventing NFL from being higher in bvFTD/FTD/FTLDSs seems to be the presence of ALS patients amongst the broad category of ONDs in several studies [40, 41, 42, 43].

In our view, the most important findings of our meta‐analysis are the following. (1) ALS is characterized by a marked increase in CSF and blood NFL levels, which differentiates it from non‐neurodegenerative conditions but also from most other chronic neurodegenerative diseases. (2) In contrast, NFL (both in CSF and in blood) is not as good at discriminating between FTD and other dementias, and this is particularly true in the case of bvFTD, in which NFL levels are generally lower than in nfvPPA and especially svPPA. Therefore, NFL measurement is unlikely to be helpful as a single instrument for the highly needed diagnostic discrimination between bvFTD and AD. (3) Nevertheless, our findings may indirectly support the diagnostic utility of NFL in the differentiation between bvFTD and primary psychiatric disorders, for which promising data have been provided by single investigations [16, 32, 38], as the latter category is included in our meta‐analysis in the group of NHCs. (4) The strong similarity, at least at the qualitative level, observed in our analyses between the findings on NFL levels in CSF and in blood highlights the biological–clinical value of NFL measurement in blood as a less invasive, but possibly equally informative, laboratory investigation. This also indirectly supports the view of blood NFL levels as reflecting CSF levels due to transfer of this protein from the CSF space to the bloodstream [9].

At the same time, it is acknowledged that our work has the following limitations. (1) Amongst analysed studies on blood NFL, no differentiation was made between those in which the analyte was measured in serum and those in which it was measured in plasma. (2) Investigations were not separated on the basis of the method used to quantify NFL (e.g., enzyme‐linked immunosorbent assay vs. single molecule array [Simoa]). (3) Regarding studies on ALS, our classification of certain non‐ALS categories as AMs or as ONDs was based on what was reported by the authors of the studies, but certain disease entities may be classified as AMs in some studies and as ONDs in others. Although this hinders a rational subdivision of controls for meta‐analytic purposes and therefore may reduce the informativity of our results, it is still considered to be the least biased implementable strategy, as clinical details about individual patients in those categories are not available in all studies and a re‐categorization of patients by the authors of the meta‐analysis would have been unacceptably arbitrary. (4) As regards FTD, specific analyses on the diagnostic discrimination from individual non‐FTD dementias (e.g., AD or dementia with Lewy bodies) were not conducted, nor from patients with primary psychiatric disorders. (5) Within the category of FTD, bvFTD (as it is the most common form of FTD and because of the practical relevance of the differential diagnosis from AD and other dementias) was considered separately for further comparisons, but not the other individual entities nfvPPA and svPPA; likewise, analyses were performed regarding the broad category of FTLDSs but not its individual components other than FTD and bvFTD, namely progressive supranuclear palsy and corticobasal syndrome. This was due to the prominent motor disturbances characterizing these diseases, however, and qualifying them as atypical parkinsonian syndromes, thus belonging to the field of movement disorders rather than to that of dementia. (6) For certain comparisons, the two‐strata classification of studies was not applied because of low numbers of available investigations. In addition to overcoming some limitations of the present work (e.g., by considering primary psychiatric disorders as a separate entity to be compared to bvFTD), future meta‐analyses on the still expanding theme of NFL in ALS and FTD should also address the differentiation between the two main pathological forms of FTLD (FTLD‐tau and FTLD‐TDP) [18]. This point will have increasing importance in the future, when diverse pathology‐specific therapeutics will hopefully become available. Moreover, clinically relevant aspects other than the diagnostic one should also undergo meta‐analytic assessment, namely the prognostic value of CSF and blood NFL in both ALS and FTD [13, 17].

## AUTHOR CONTRIBUTIONS

**Federico Verde:** Conceptualization; data curation; writing – original draft; writing – review and editing. **Sara Licaj:** Data curation; formal analysis; methodology; investigation. **Davide Soranna:** Conceptualization; data curation; methodology; investigation; visualization; formal analysis. **Nicola Ticozzi:** Supervision; writing – review and editing; validation. **Vincenzo Silani:** Supervision; writing – review and editing; validation. **Antonella Zambon:** Conceptualization; data curation; formal analysis; writing – review and editing; supervision; methodology; validation.

## FUNDING INFORMATION

This work was funded by BIBLIOSAN.

## CONFLICT OF INTEREST STATEMENT

FV is Associate Editor of the *Journal of Alzheimer's Disease*. NT has received compensation for consulting services from Amylyx Pharmaceuticals and Zambon Biotech SA; he is Associate Editor of *Frontiers in Aging Neuroscience*. VS has received compensation for consulting services and/or speaking activities from AveXis, Cytokinetics, Italfarmaco, Liquidweb S.r.l. and Novartis Pharma AG, and receives or has received research support from the Italian Ministry of Health, AriSLA and E‐Rare Joint Transnational Call; he is on the Editorial Board of *Amyotrophic Lateral Sclerosis and Frontotemporal Degeneration*, *European Neurology*, *American Journal of Neurodegenerative Disease* and *Frontiers in Neurology*. The other authors report no conflicts of interests.

## Supporting information


Figure S1.

## Data Availability

Data used in this study will be made available upon reasonable request to the corresponding author.
