# Supplementary material for: Cerebrospinal fluid and blood neurofilament light chain levels in amyotrophic lateral sclerosis and frontotemporal degeneration: A meta‐analysis
Source: Eur J Neurol. 2024 Jun 27;31(9):e16371. doi: 10.1111/ene.16371 (PMC11295179; doi:10.1111/ene.16371)
Supplement: Supplementary file 1 — Figure S1. [file ENE-31-e16371-s001.docx]

**Supplementary material**

**Figure S1**. PRISMA flowchart for studies regarding ALS.

**
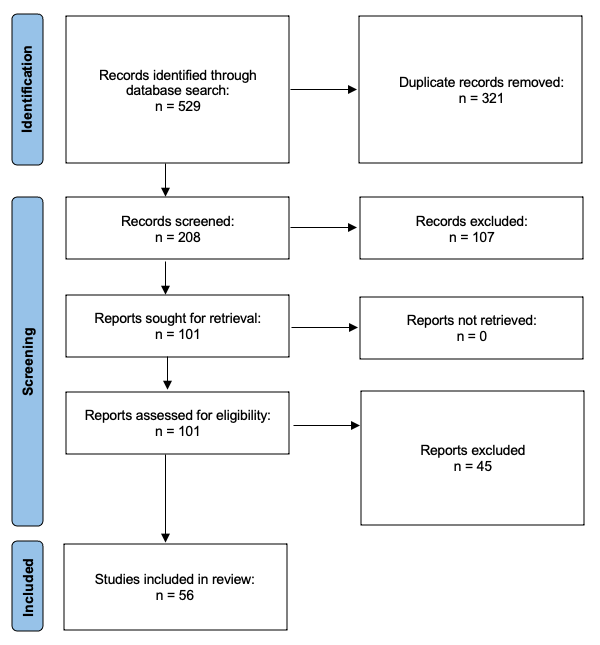
**

**Figure S2**. PRISMA flowchart for studies regarding FTD and related entities.


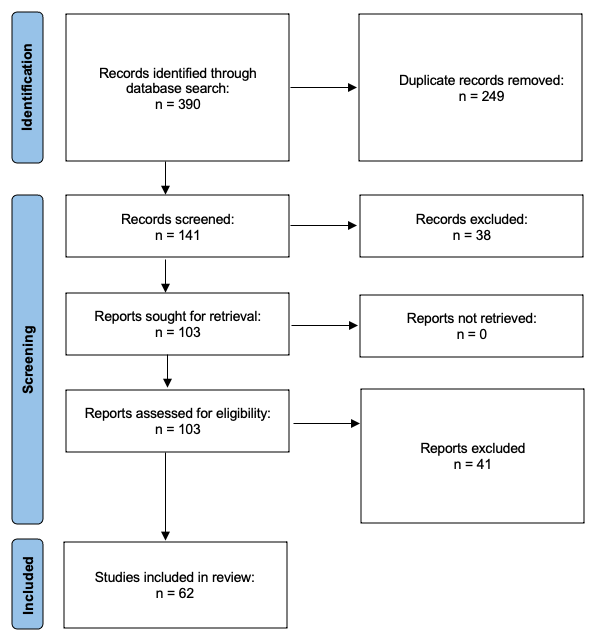


**Figure S3**. Forest plots regarding CSF and blood NFL in ALS vs. NHCs.

| Panel A: CSF |
| --- |
| 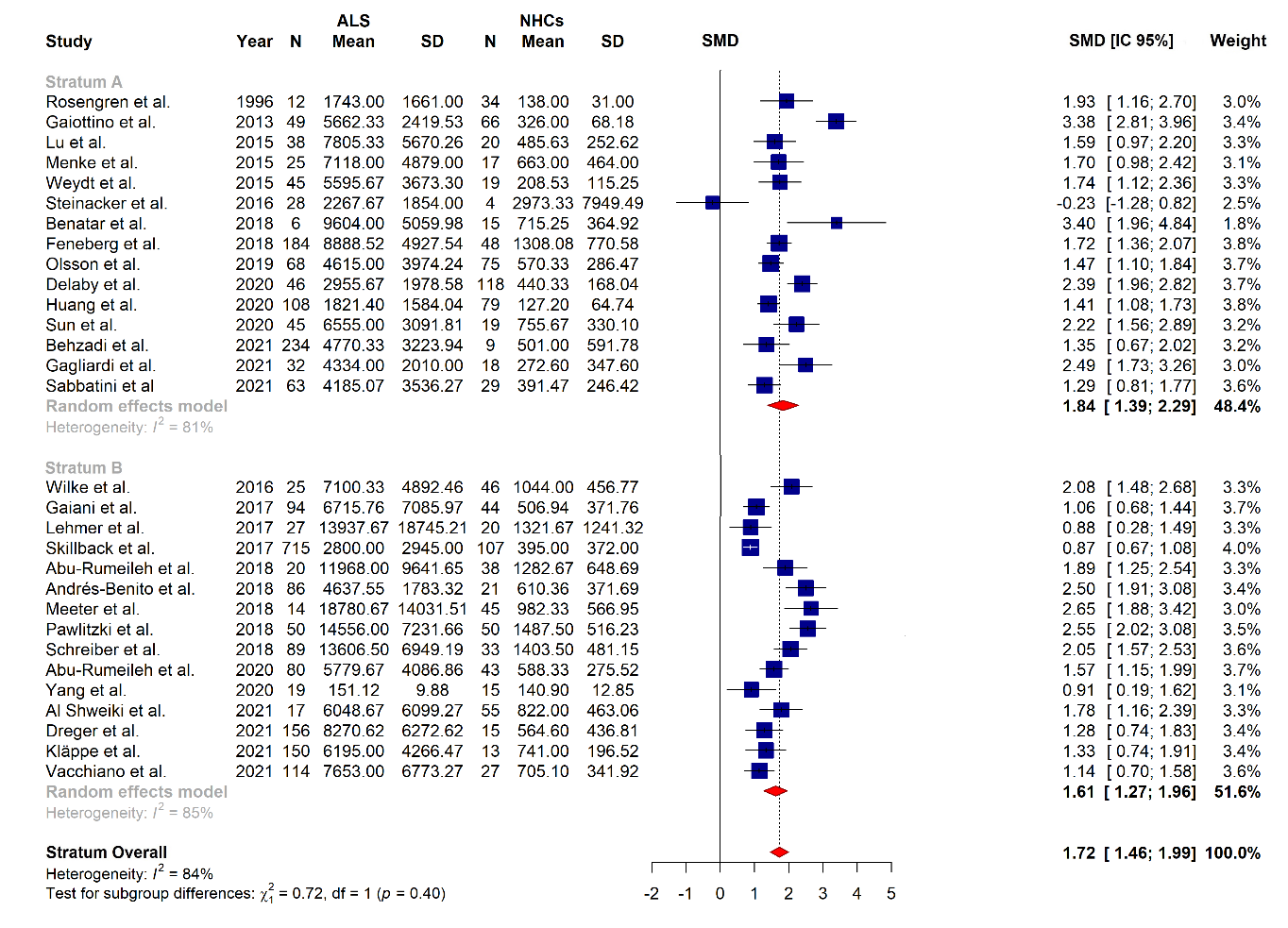 |
| Panel B: Blood |
| 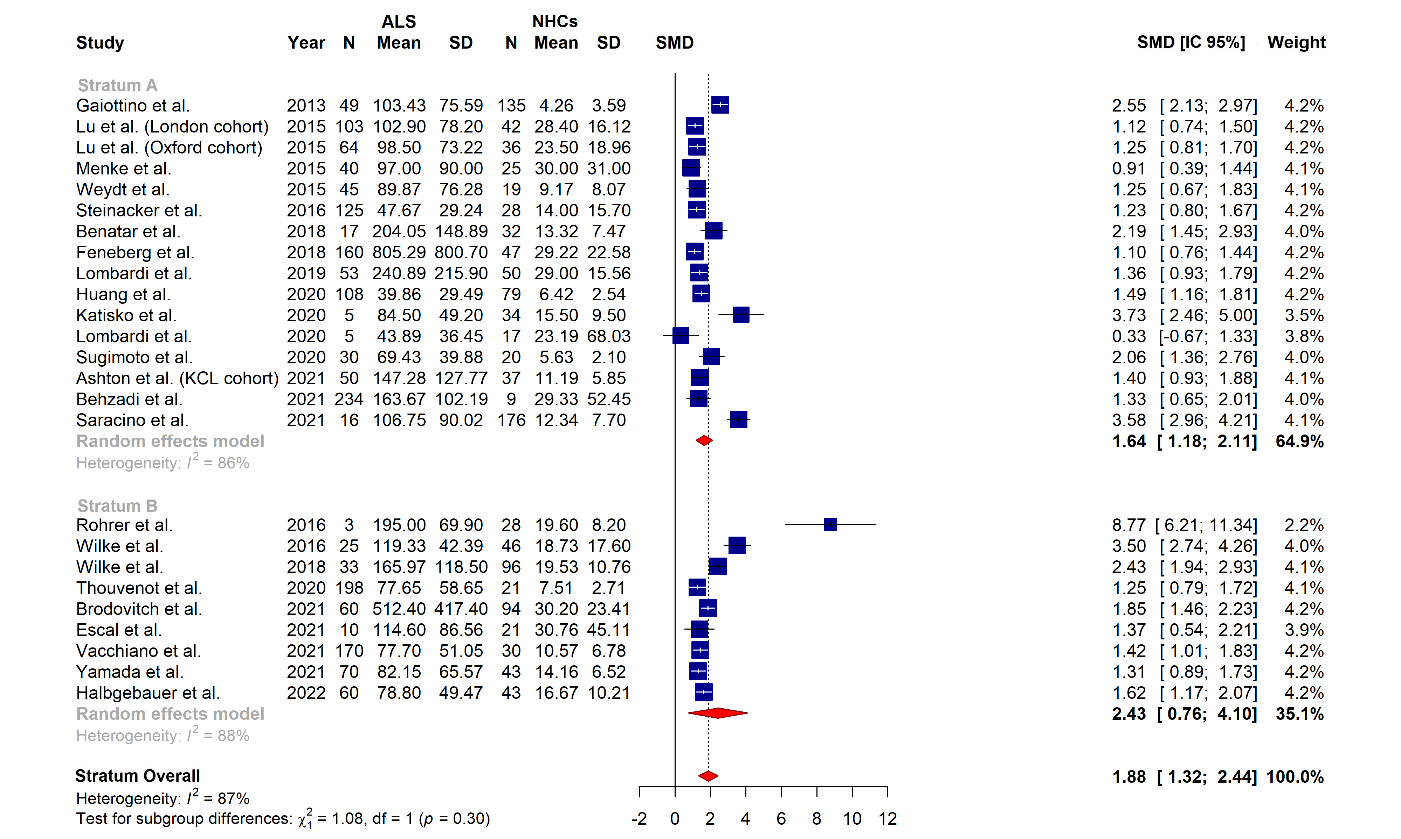 |

**Figure S4**. Forest plots regarding CSF and blood NFL in ALS vs. AMs.

| Panel A: CSF |
| --- |
| 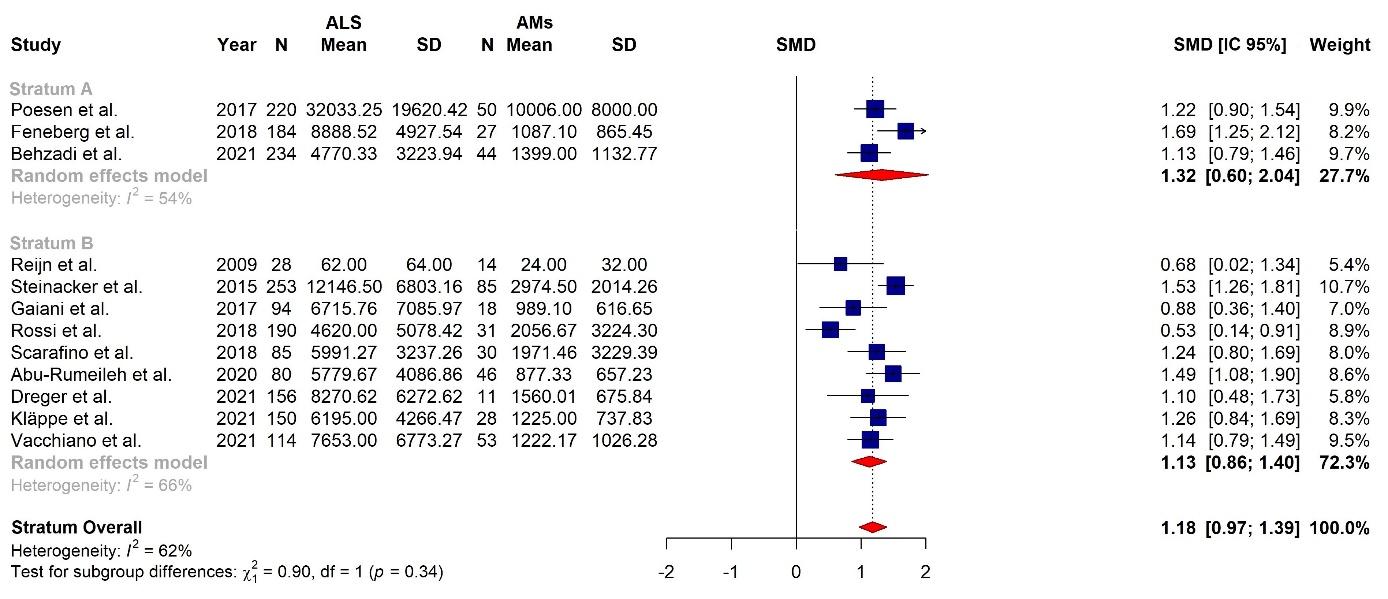 |
| Panel B: Blood |
| 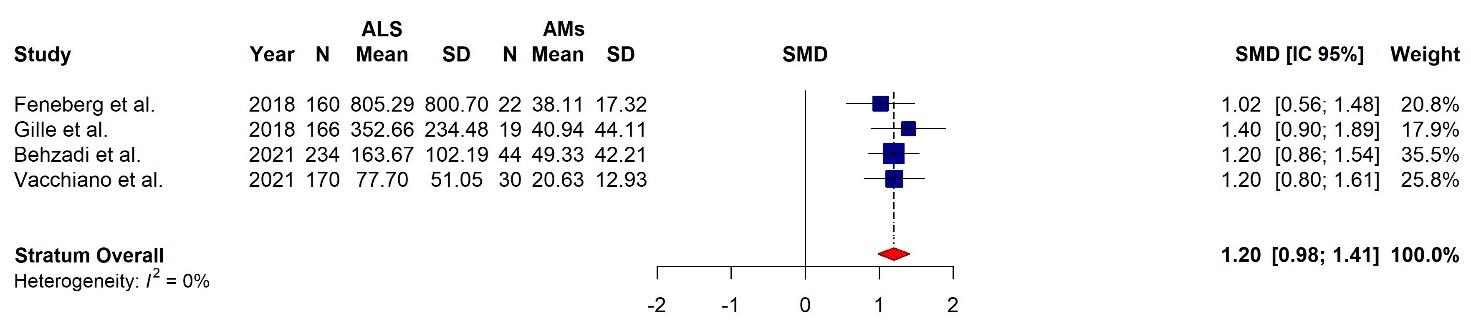 |

**Figure S5**. Forest plots regarding CSF and blood NFL in ALS vs. ONDs.

| Panel A: CSF |
| --- |
| 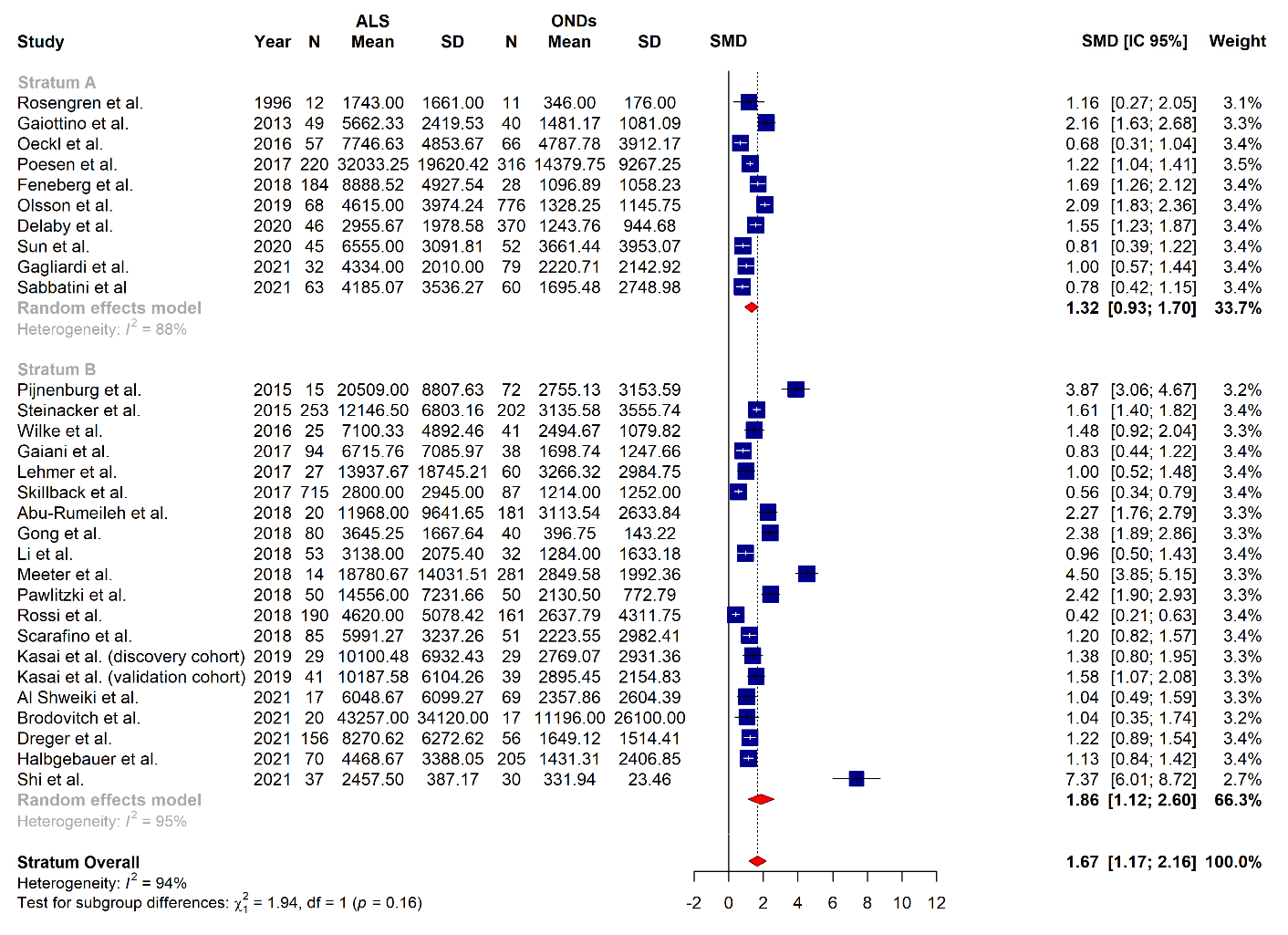 |
| Panel B: Blood |
| 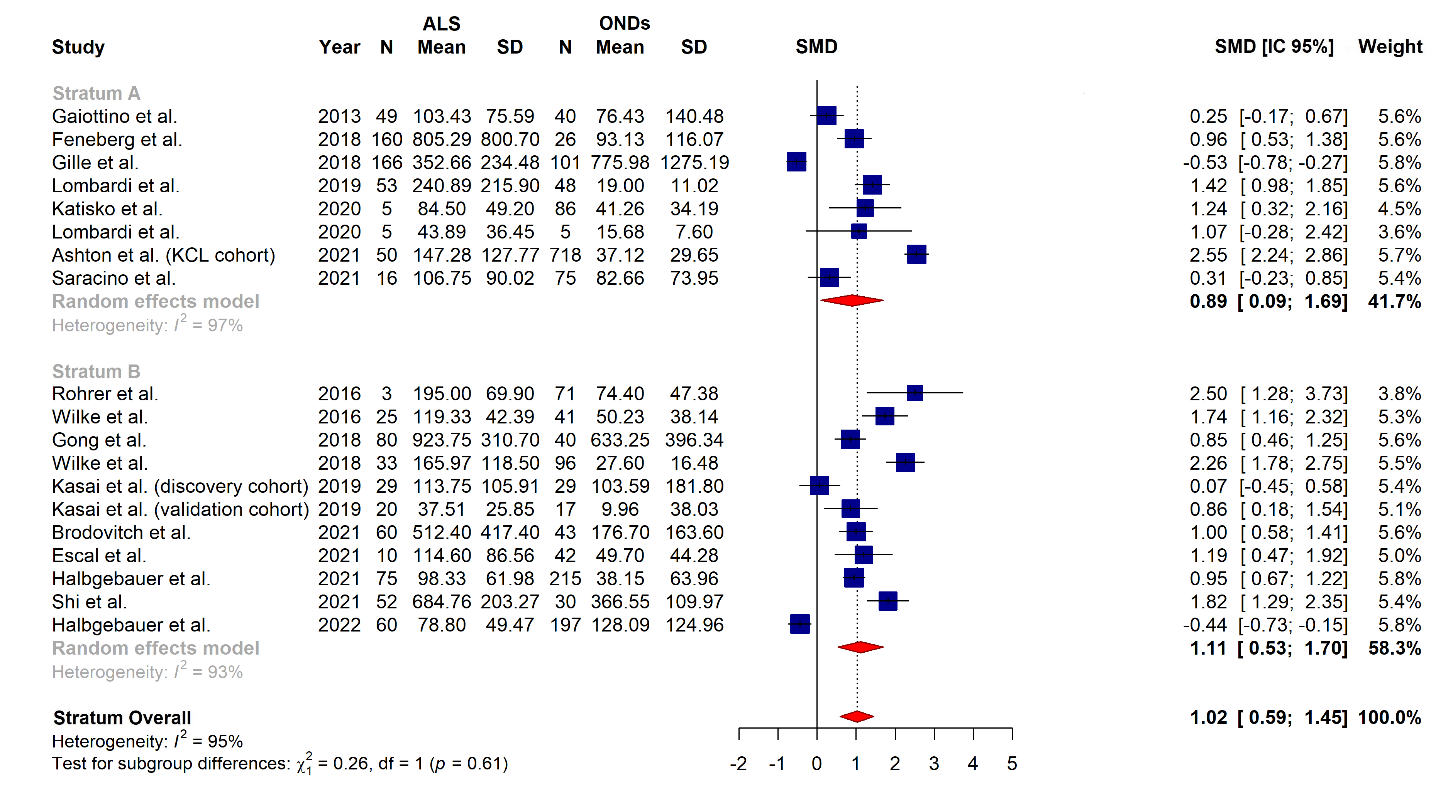 |

**Figure S6**. Forest plots regarding CSF and blood NFL in bvFTD vs. NHCs.

| Panel A: CSF |
| --- |
| 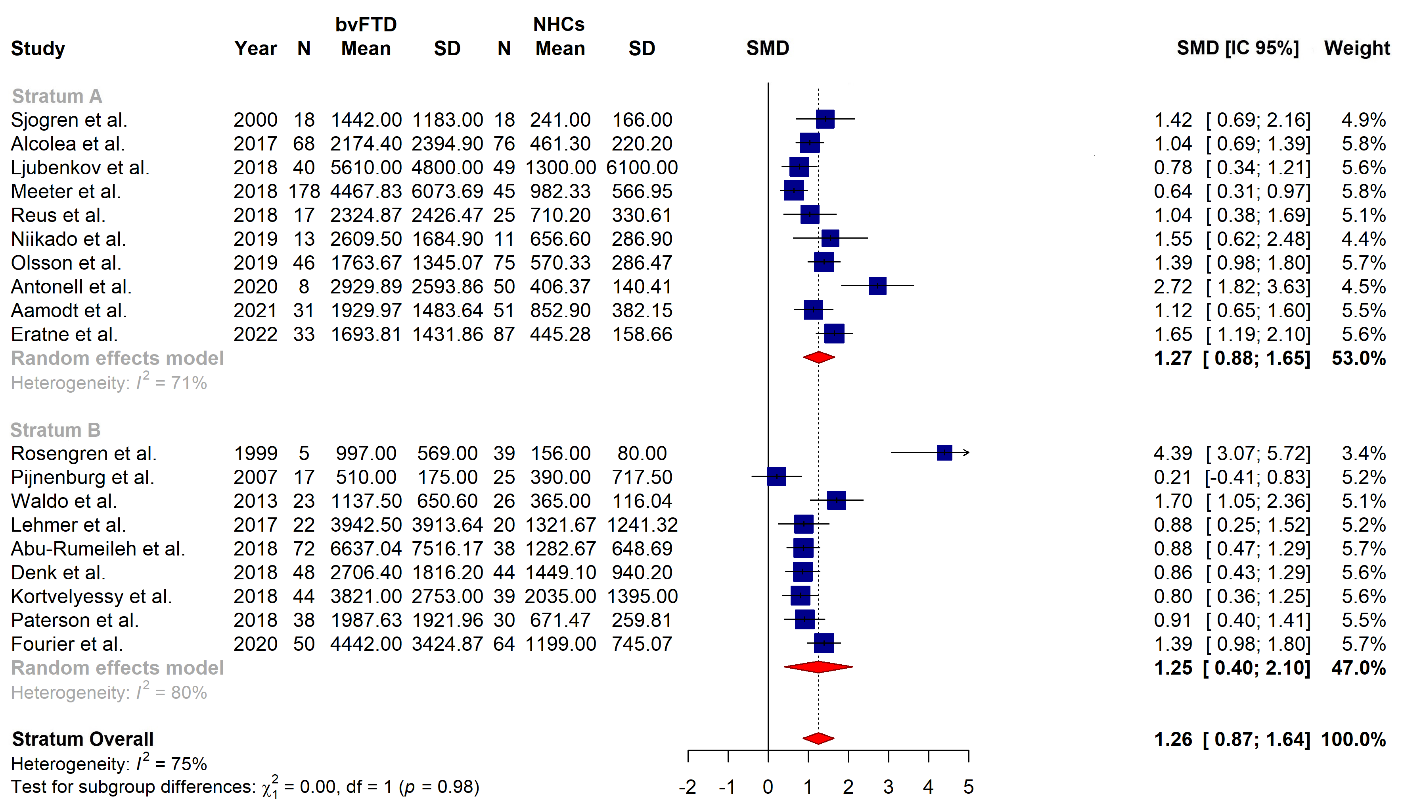 |
| Panel B: Blood |
| 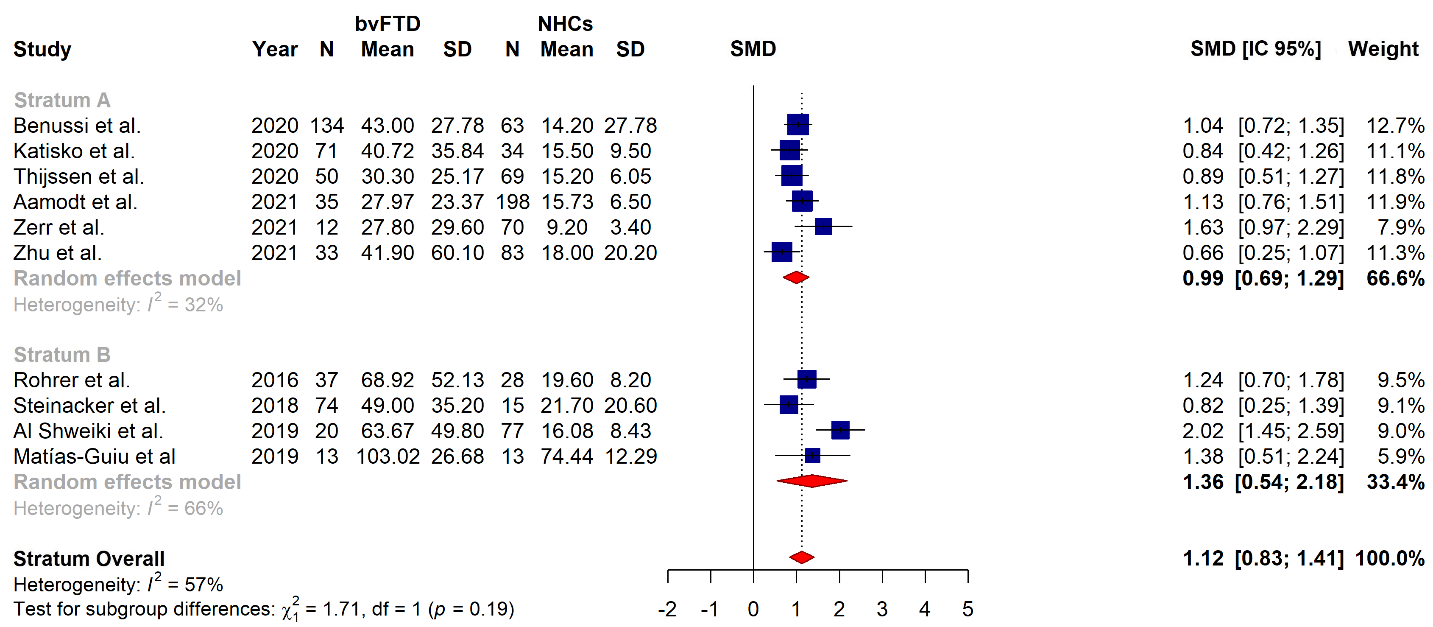 |

**Figure S7**. Forest plots regarding CSF and blood NFL in bvFTD vs. ODs.

| Panel A: CSF |
| --- |
| 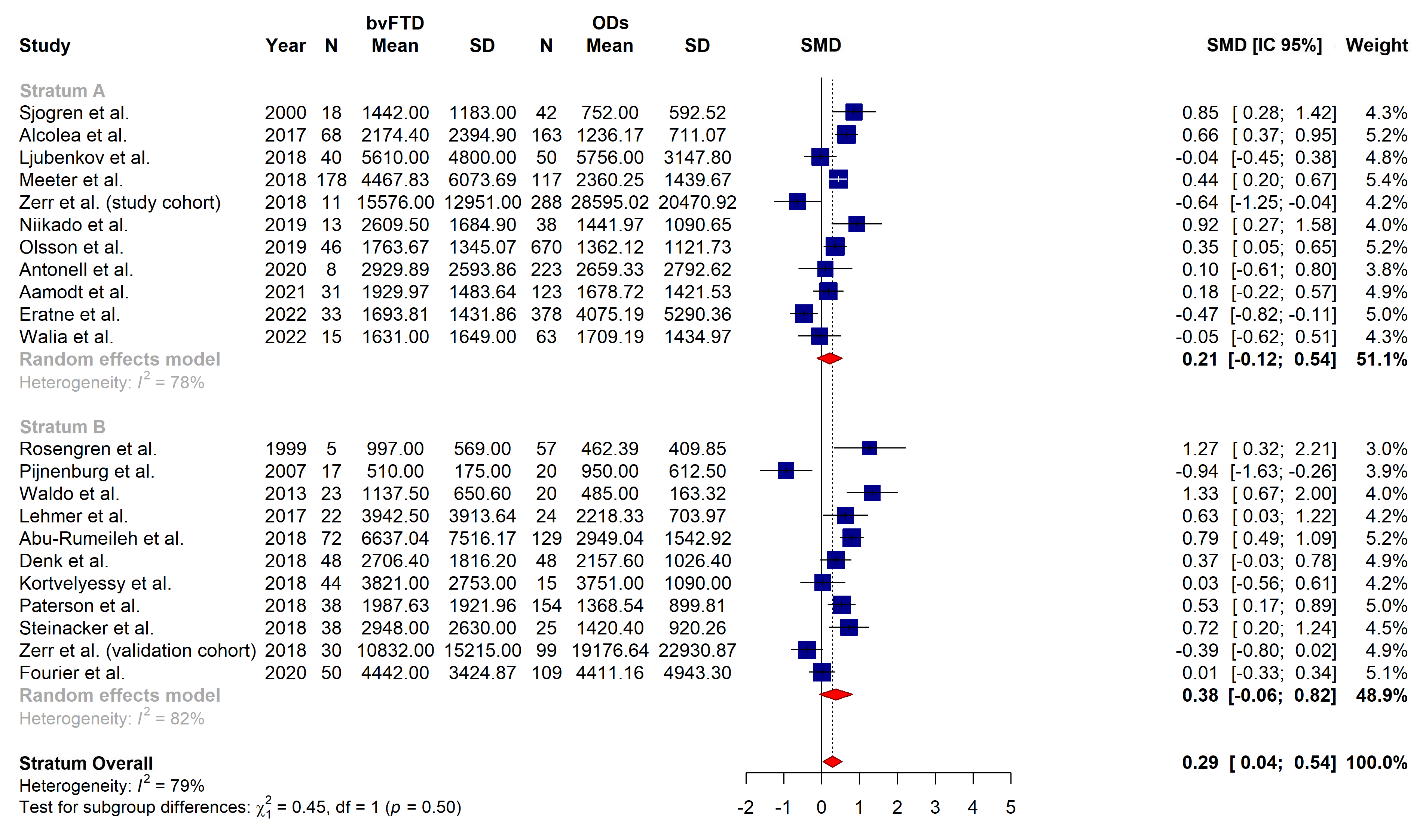 |
| Panel B: Blood |
| 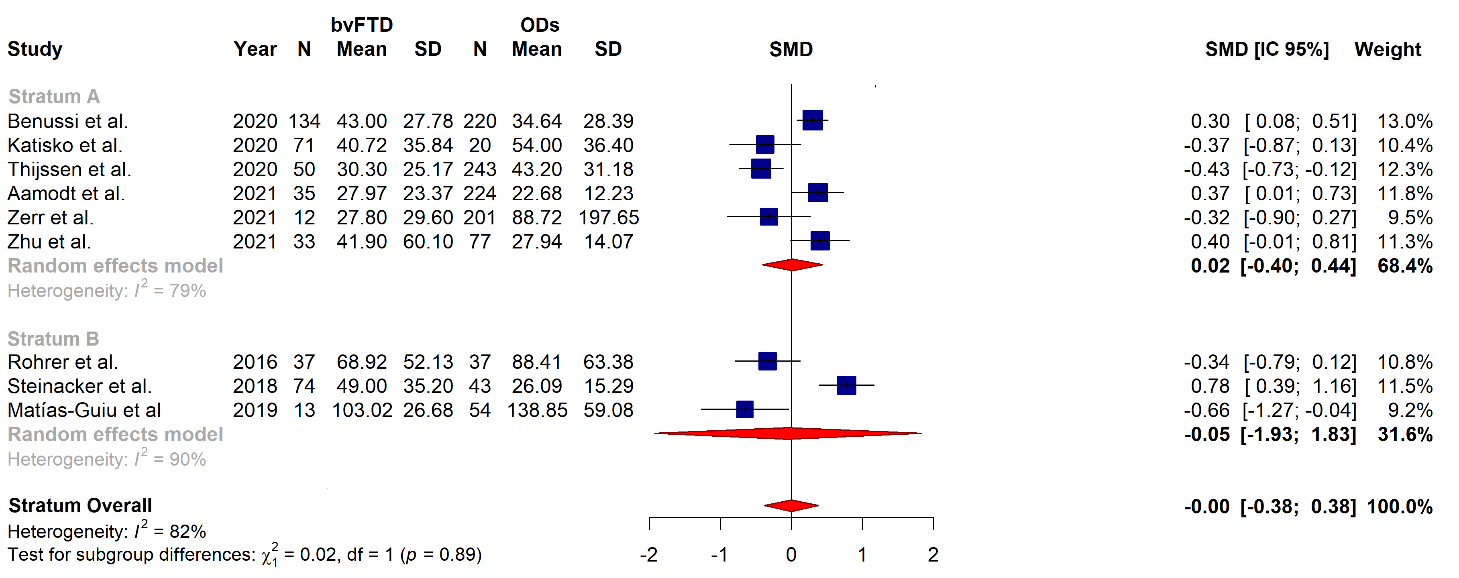 |

**Figure S8**. Forest plots regarding CSF NFL in bvFTD vs. ONDs.

| 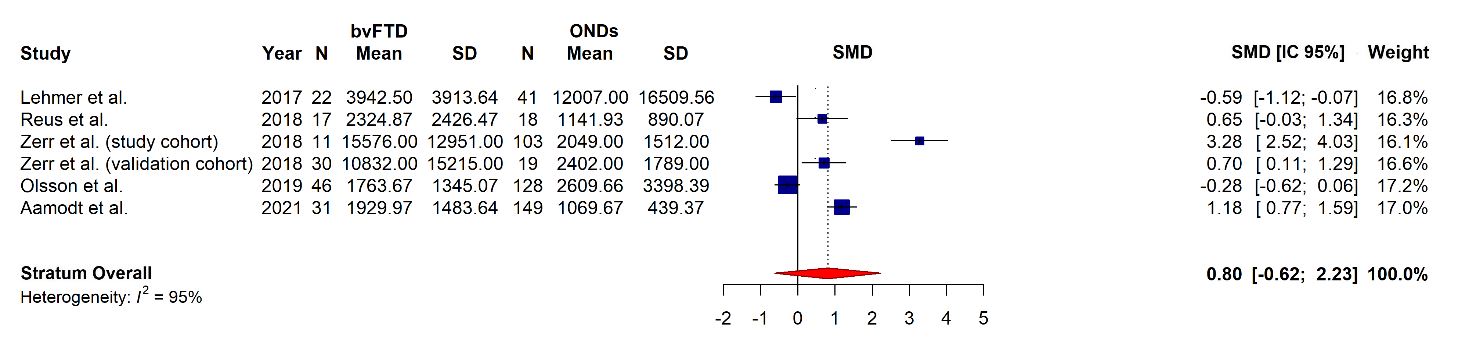 |
| --- |

**Figure S9**. Forest plots regarding CSF and blood NFL in FTD vs. NHCs.

| Panel A: CSF |
| --- |
| 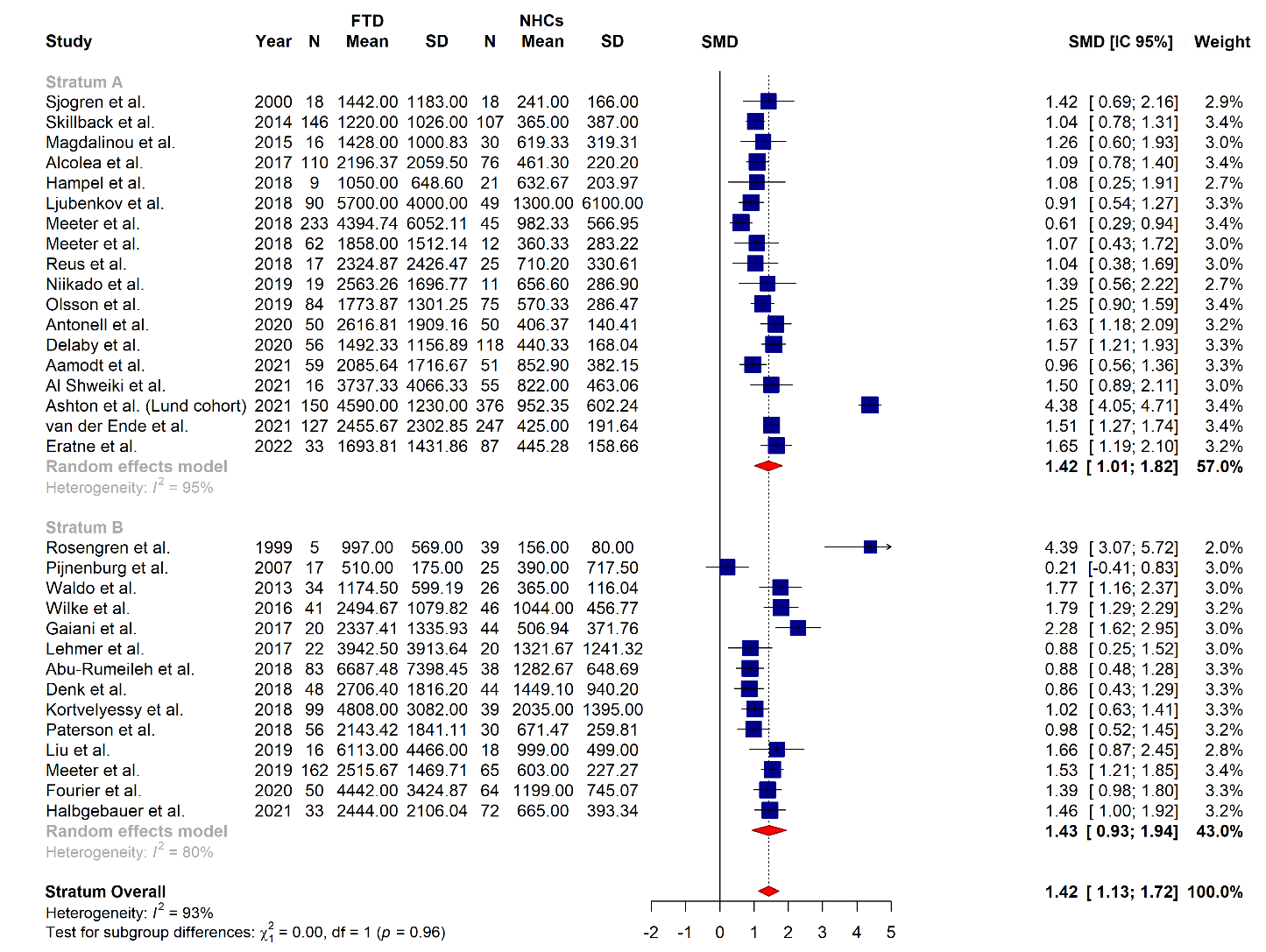 |
| Panel B: Blood |
| 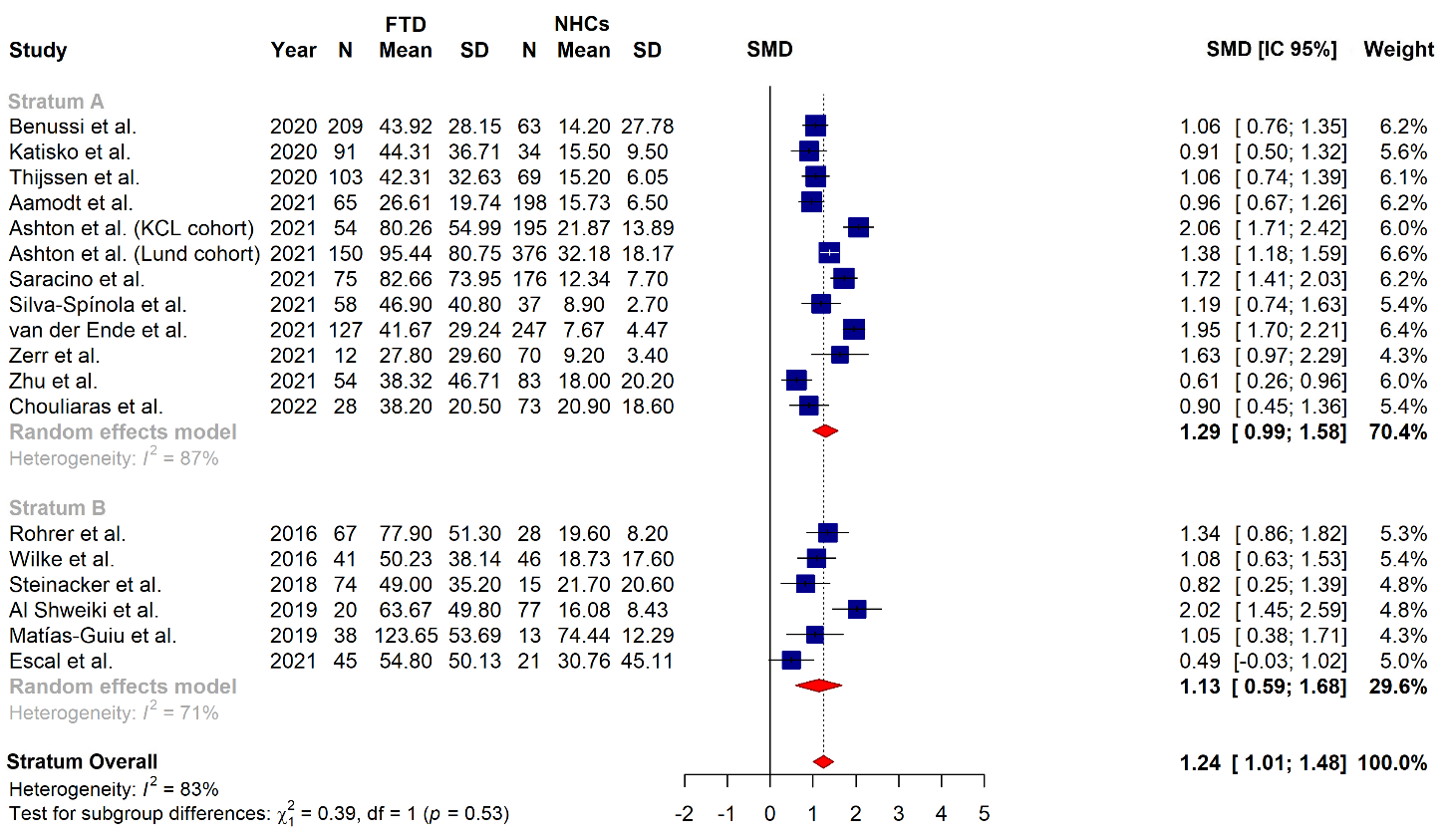 |

**Figure S10**. Forest plots regarding CSF and blood NFL in FTD vs. ODs.

| Panel A: CSF |
| --- |
| 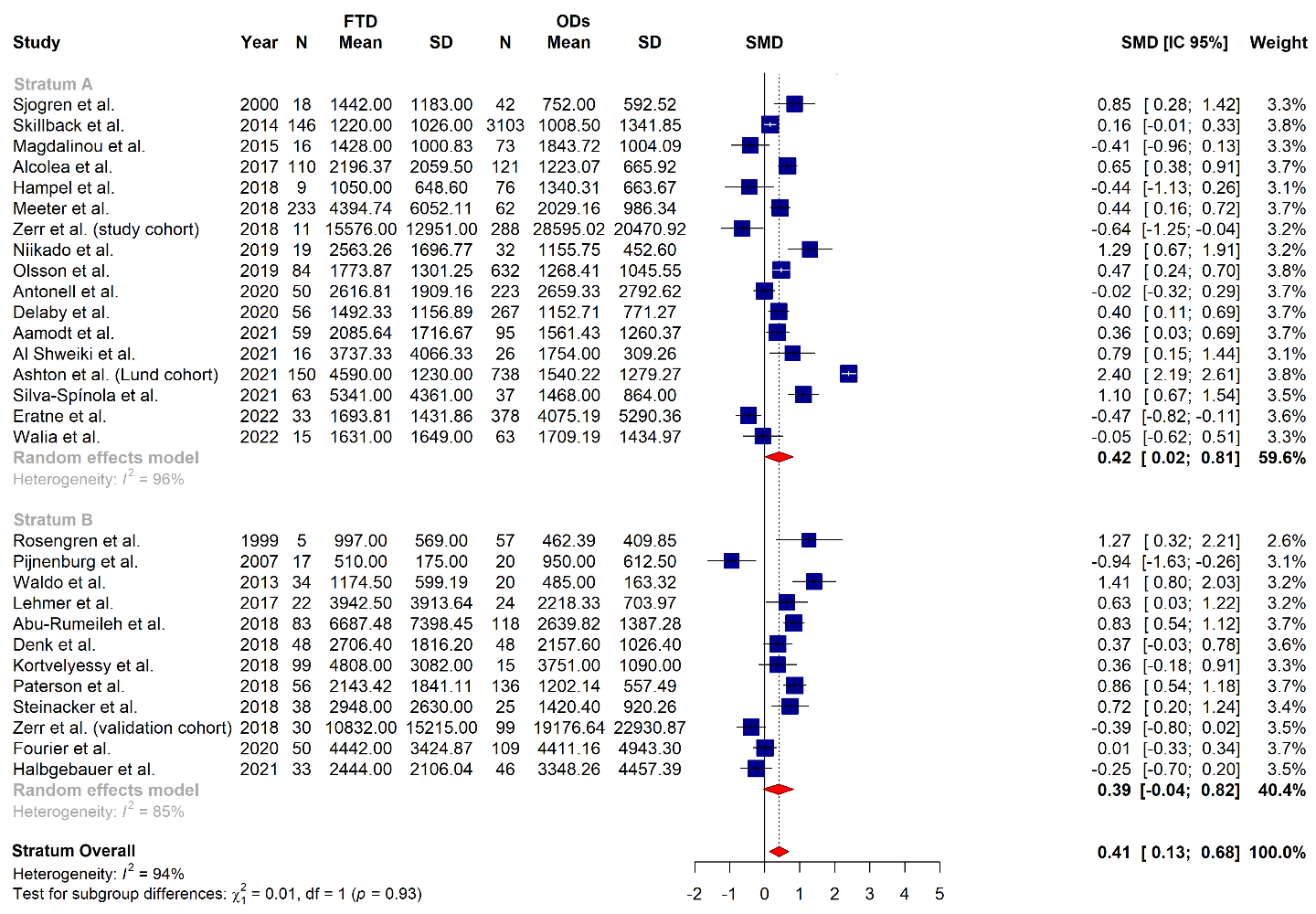 |
| Panel B: Blood |
| 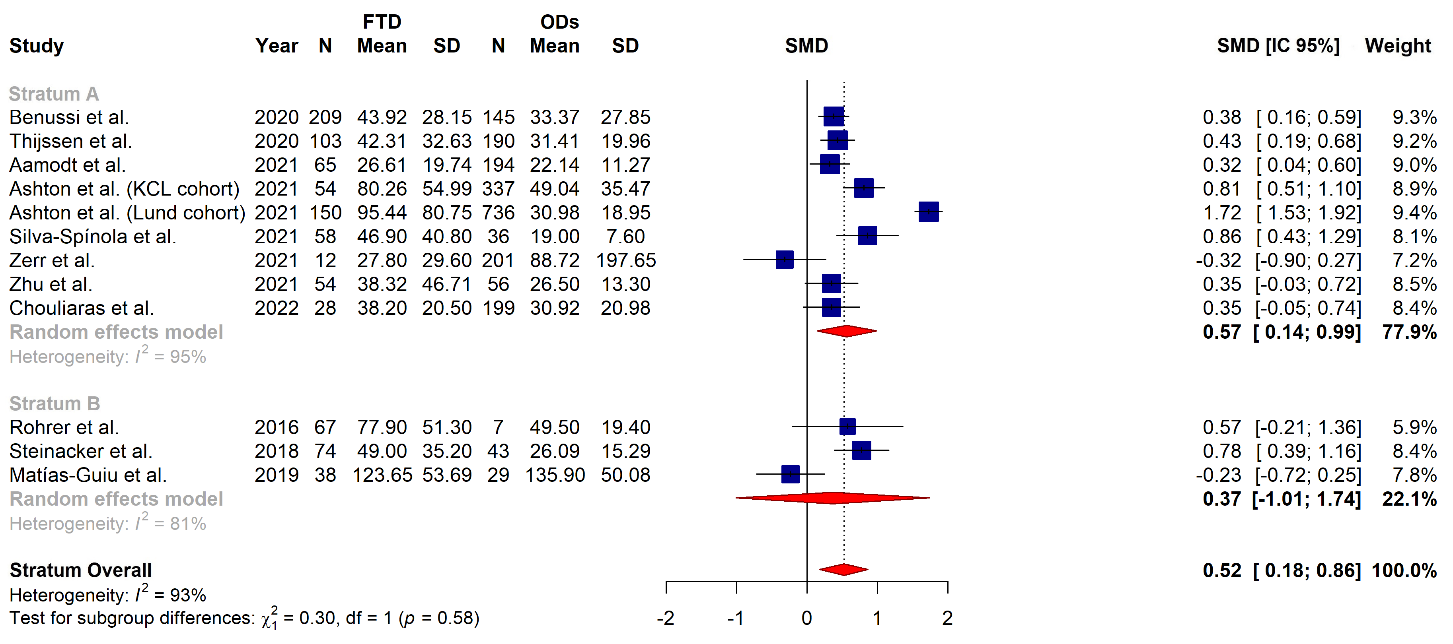 |

**Figure S11**. Forest plots regarding CSF and blood NFL in FTD vs. ONDs.

| Panel A: CSF |
| --- |
| 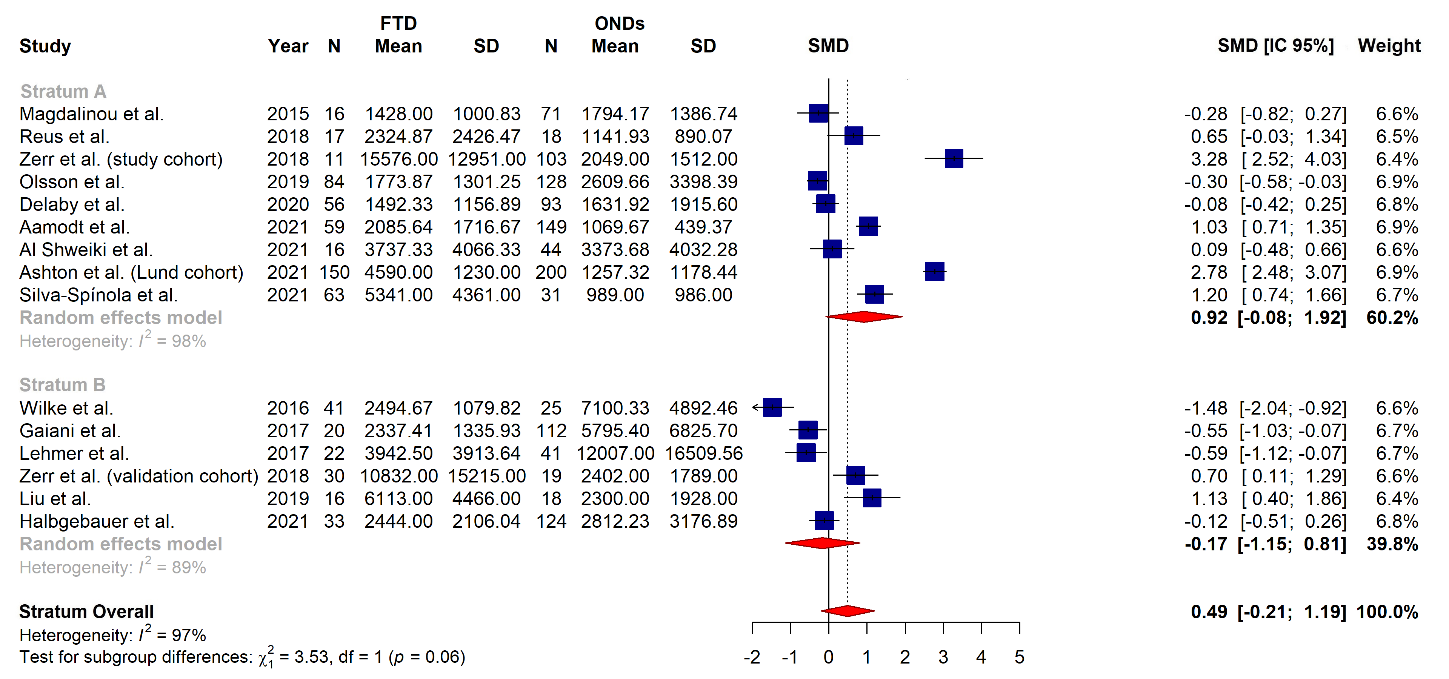 |
| Panel B: Blood |
| 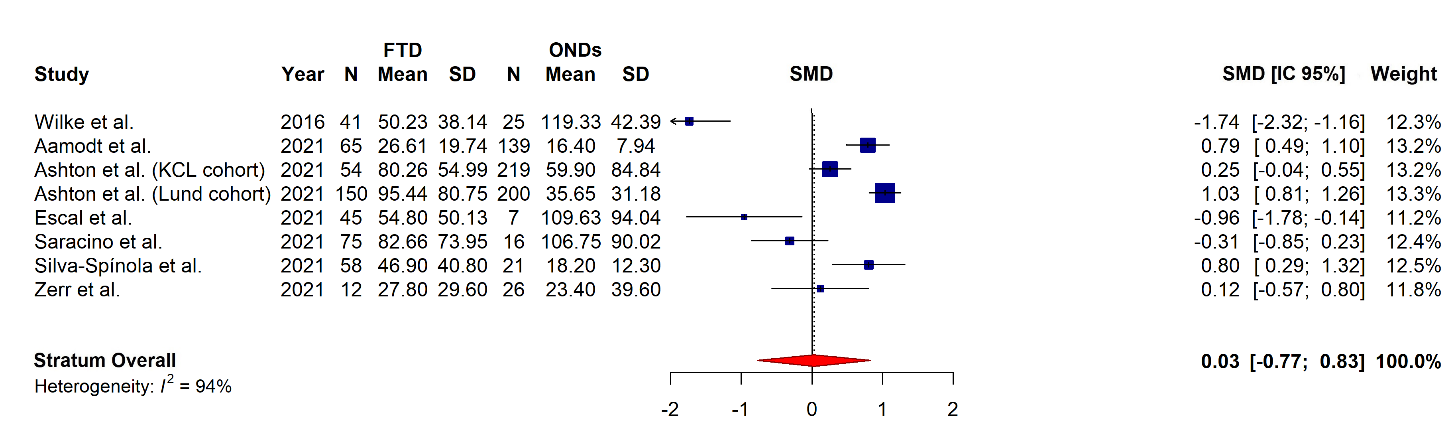 |

**Figure S12**. Forest plots regarding CSF and blood NFL in FTLDSs vs. NHCs.

| Panel A: CSF |
| --- |
| 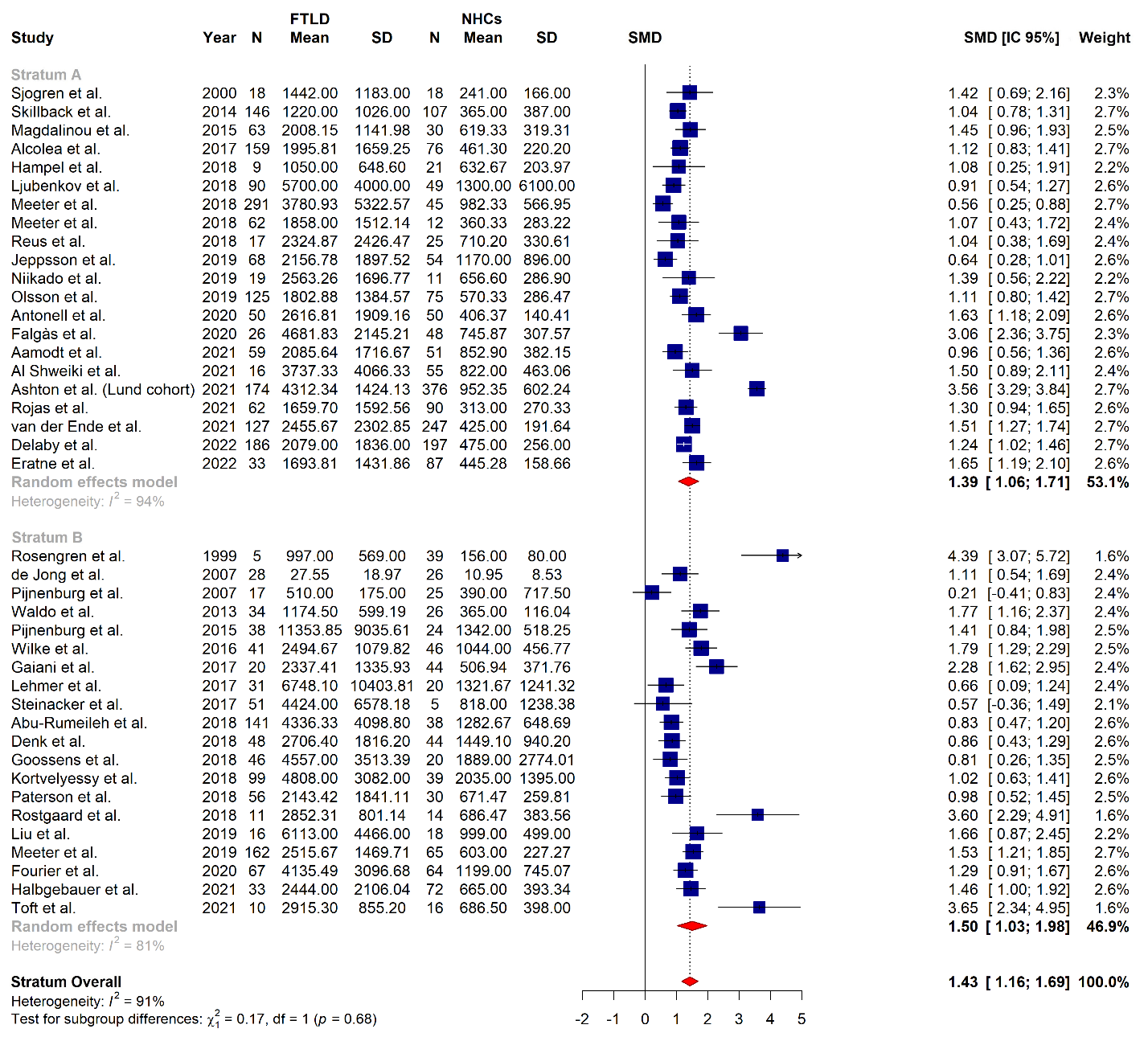 |
| Panel B: Blood |
| 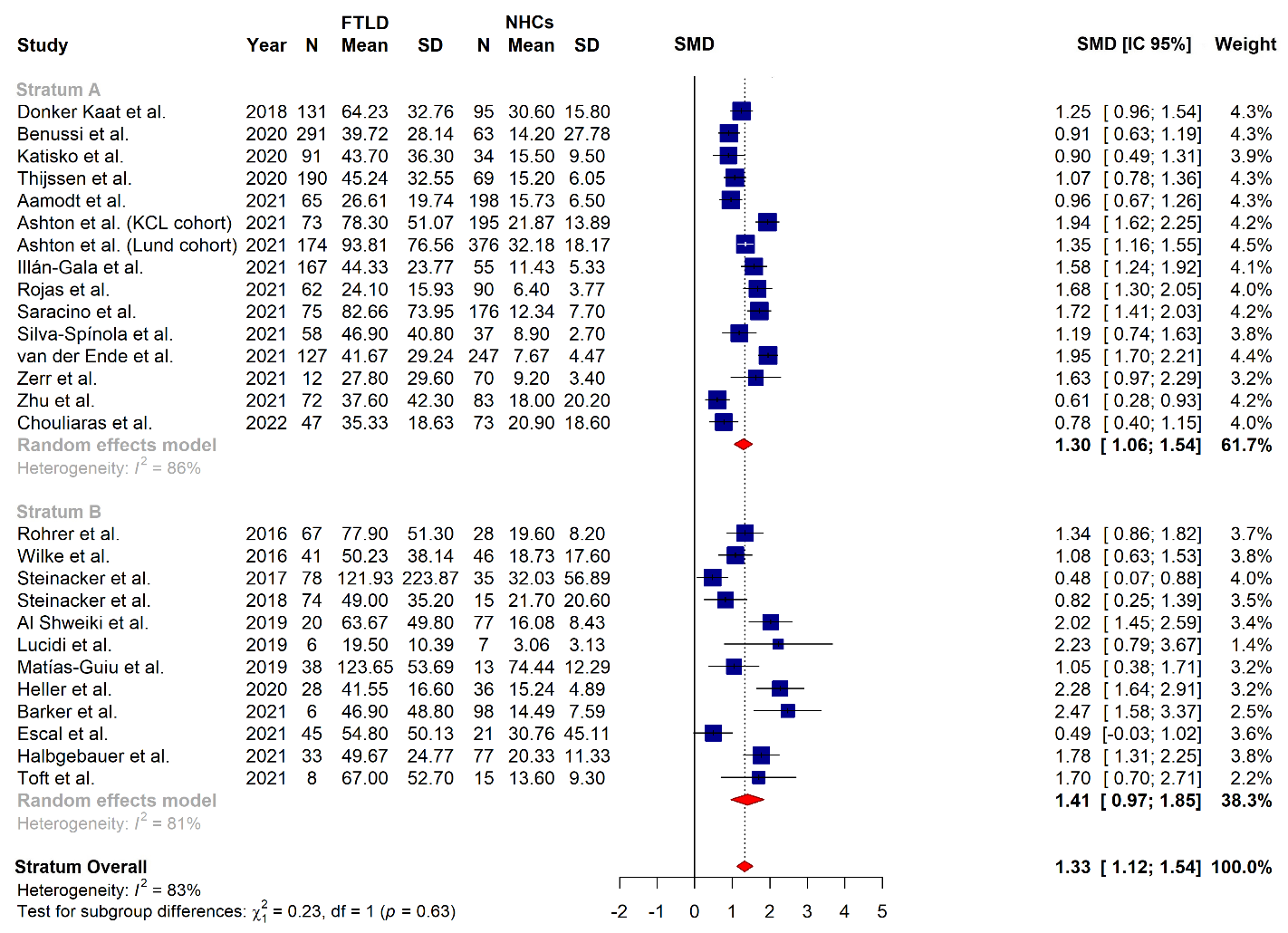 |

**Figure S13**. Forest plots regarding CSF and blood NFL in FTLDSs vs. ODs.

| Panel A: CSF |
| --- |
| 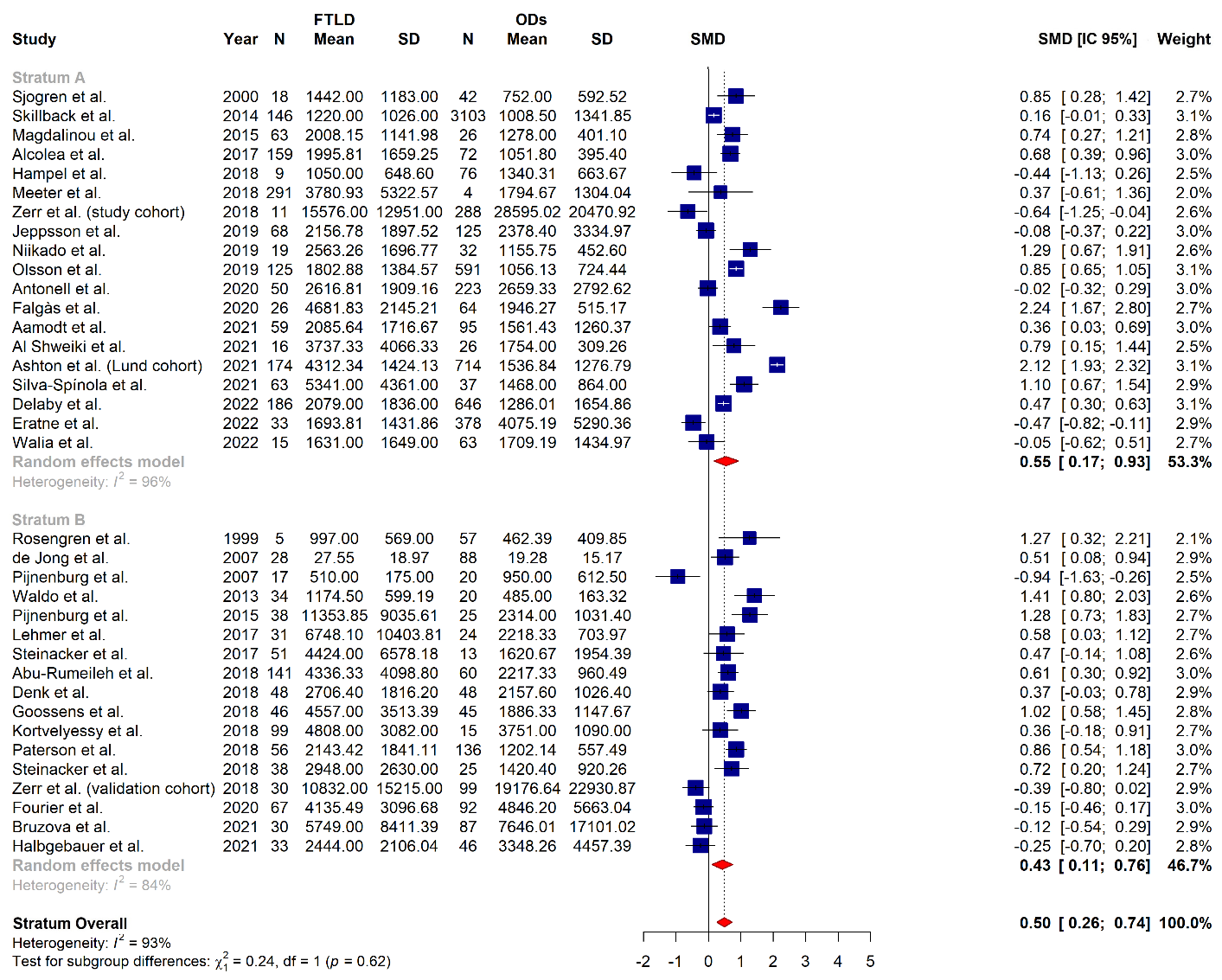 |
| Panel B: Blood |
| 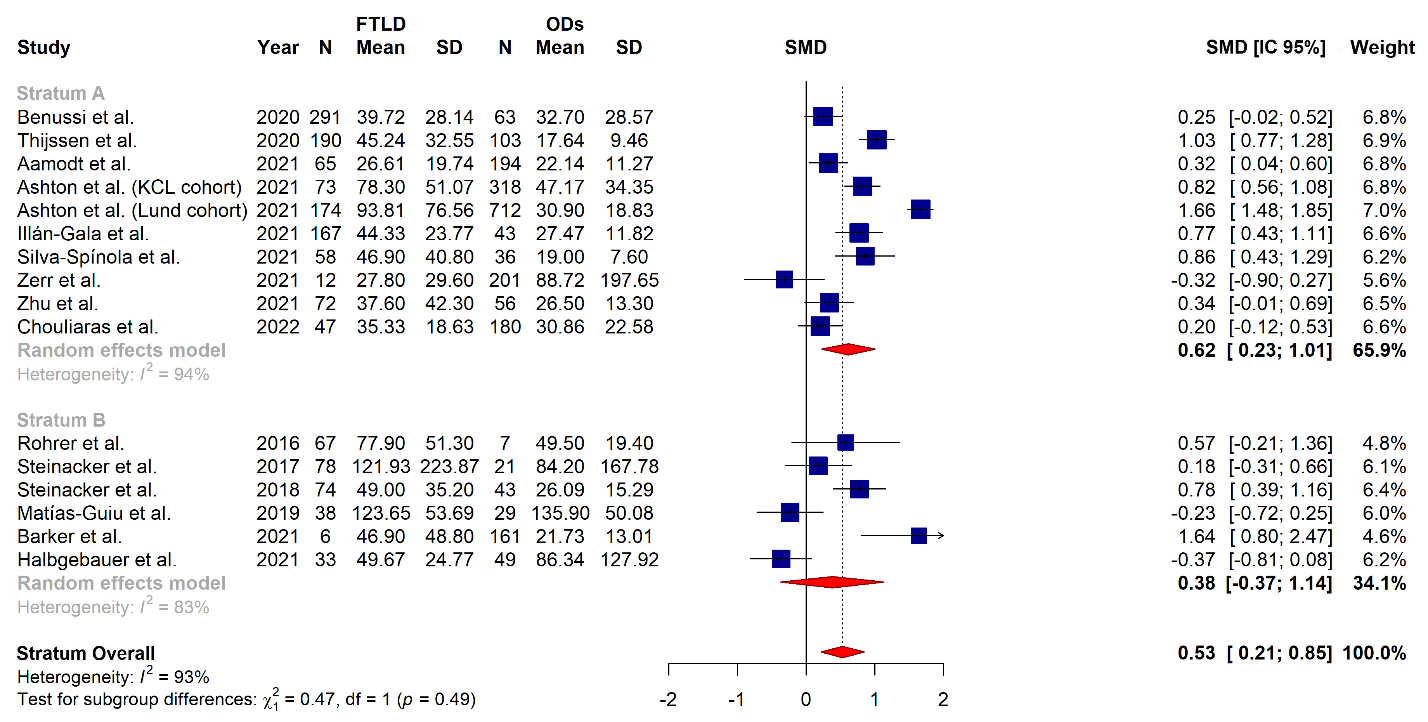 |

**Figure S14**. Forest plots regarding CSF and blood NFL in FTLDSs vs. ONDs.

| Panel A: CSF |
| --- |
| 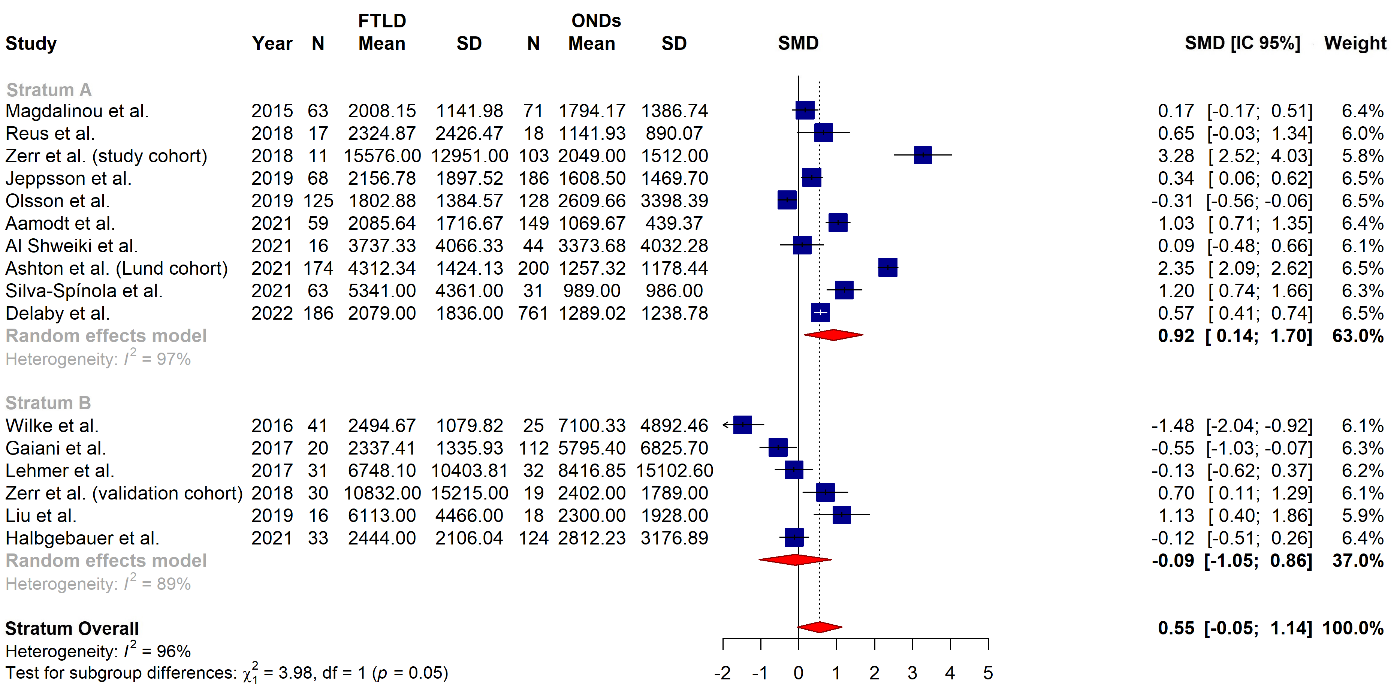 |
| Panel B: Blood |
| 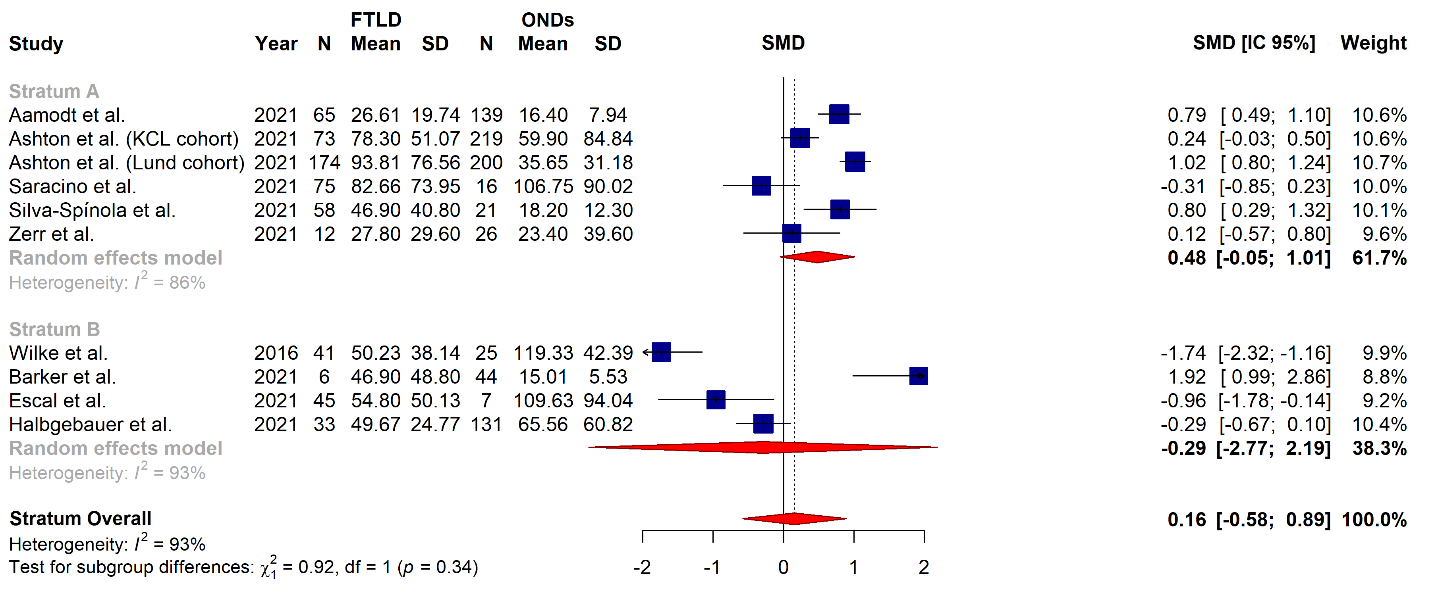 |

**Figure S15**. Influence analysis for the comparison of CSF and blood NFL levels in ALS vs. NHCs.

| Panel A: CSF |
| --- |
| 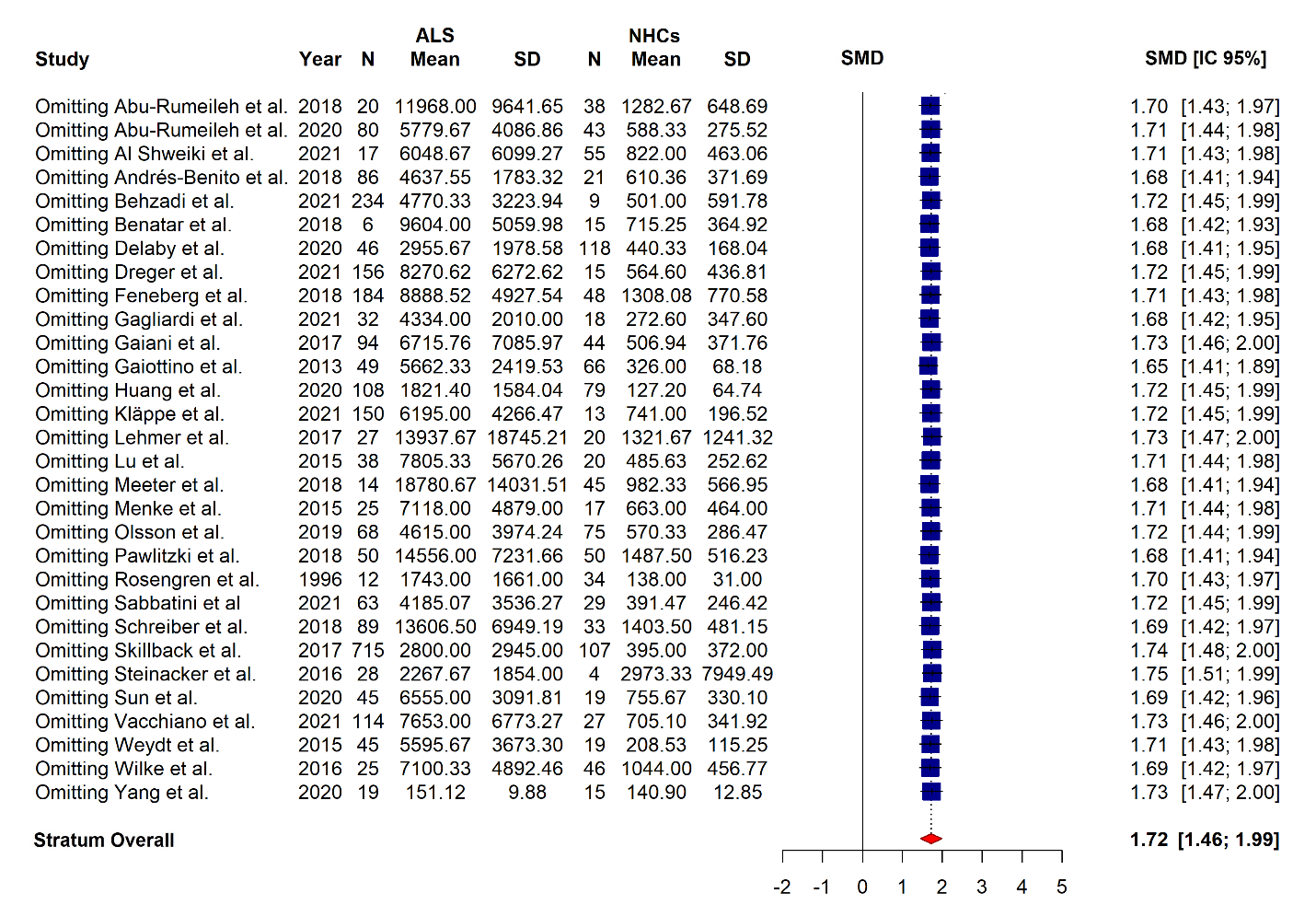 |
| Panel B: Blood |
| 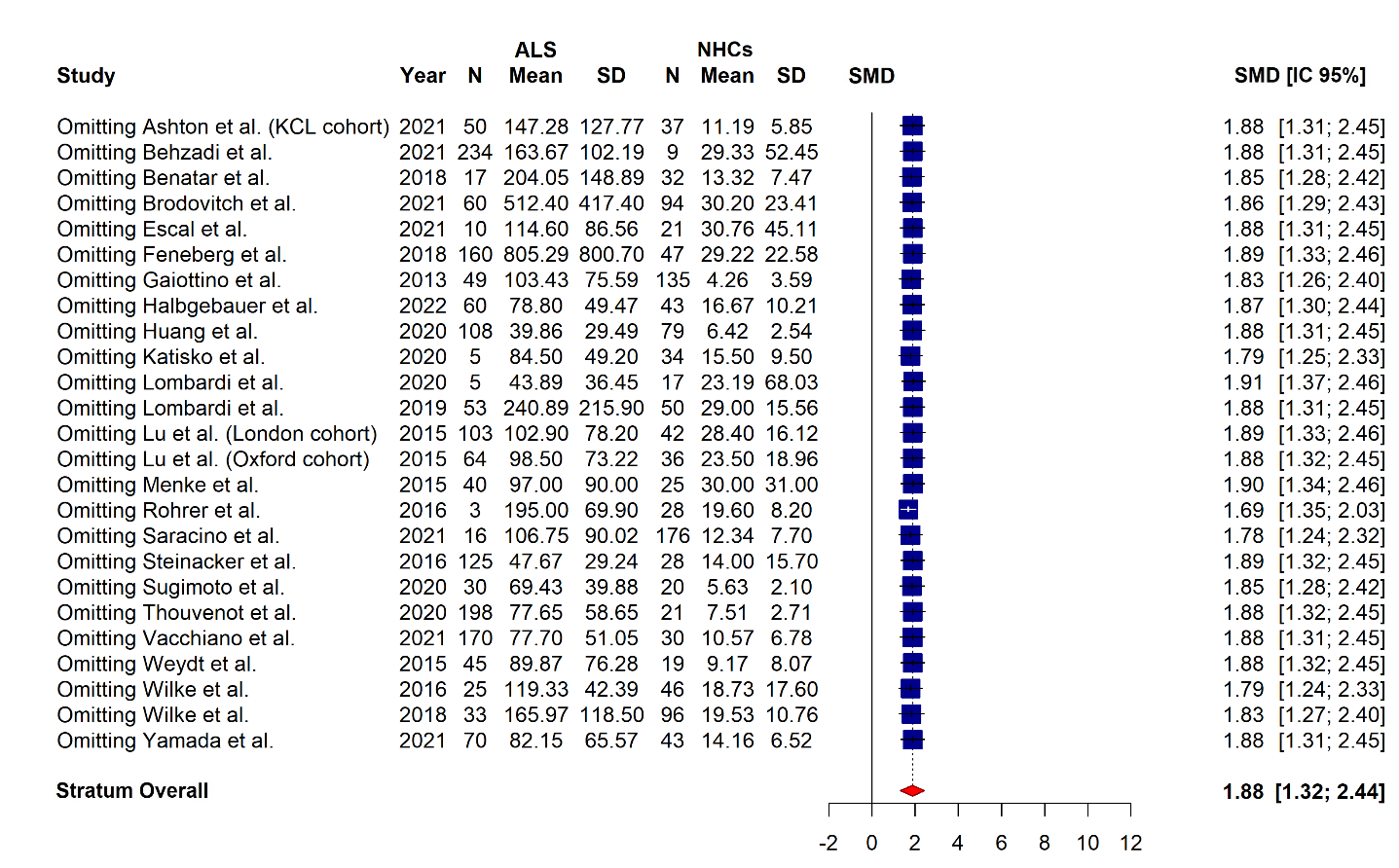 |

**Figure S16**. Influence analysis for the comparison of CSF and blood NFL levels in ALS vs. AMs.

| Panel A: CSF |
| --- |
| 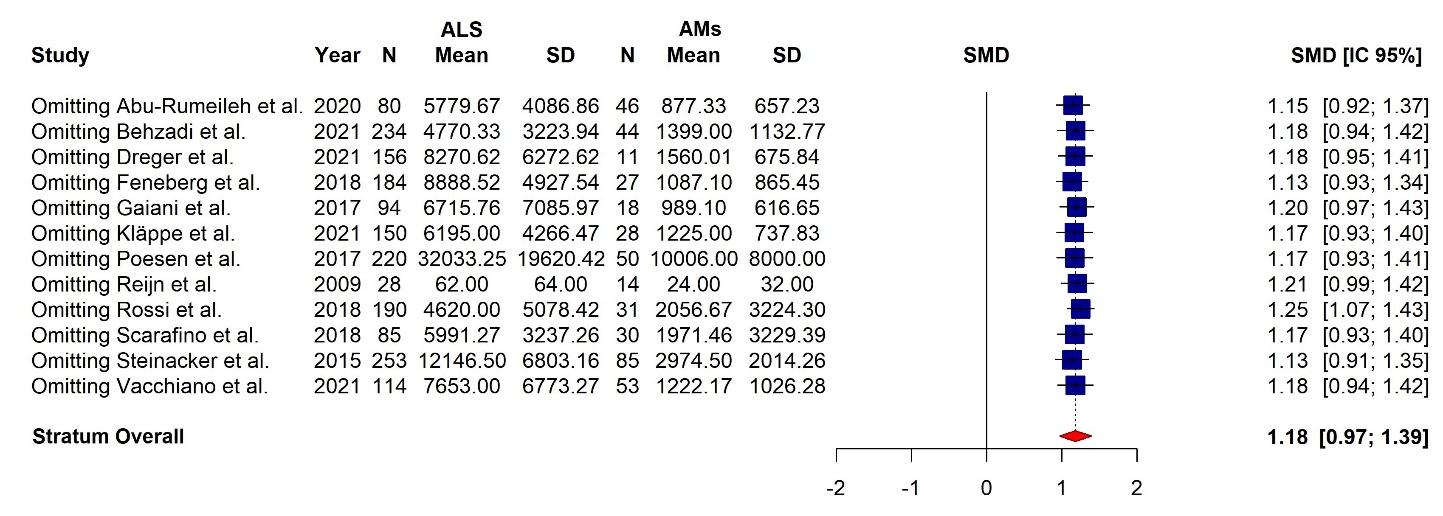 |
| Panel B: Blood |
| 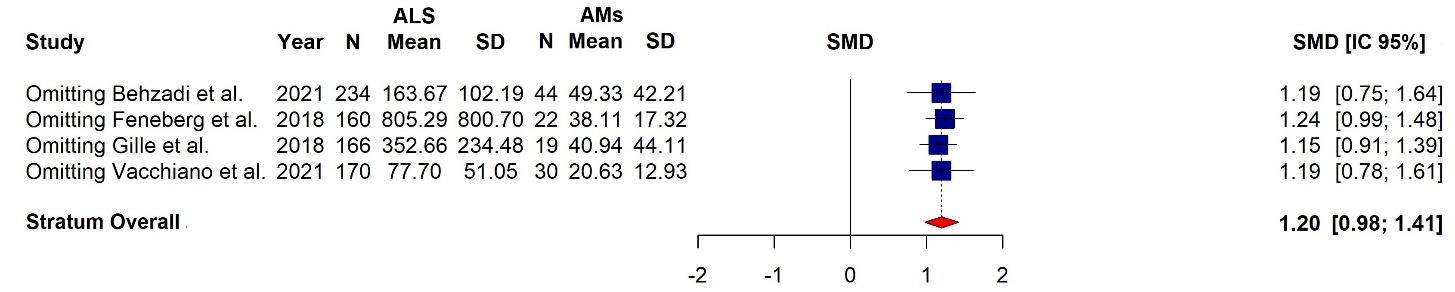 |

**Figure S17**. Influence analysis for the comparison of CSF and blood NFL levels in ALS vs. ONDs.

| Panel A: CSF |
| --- |
| 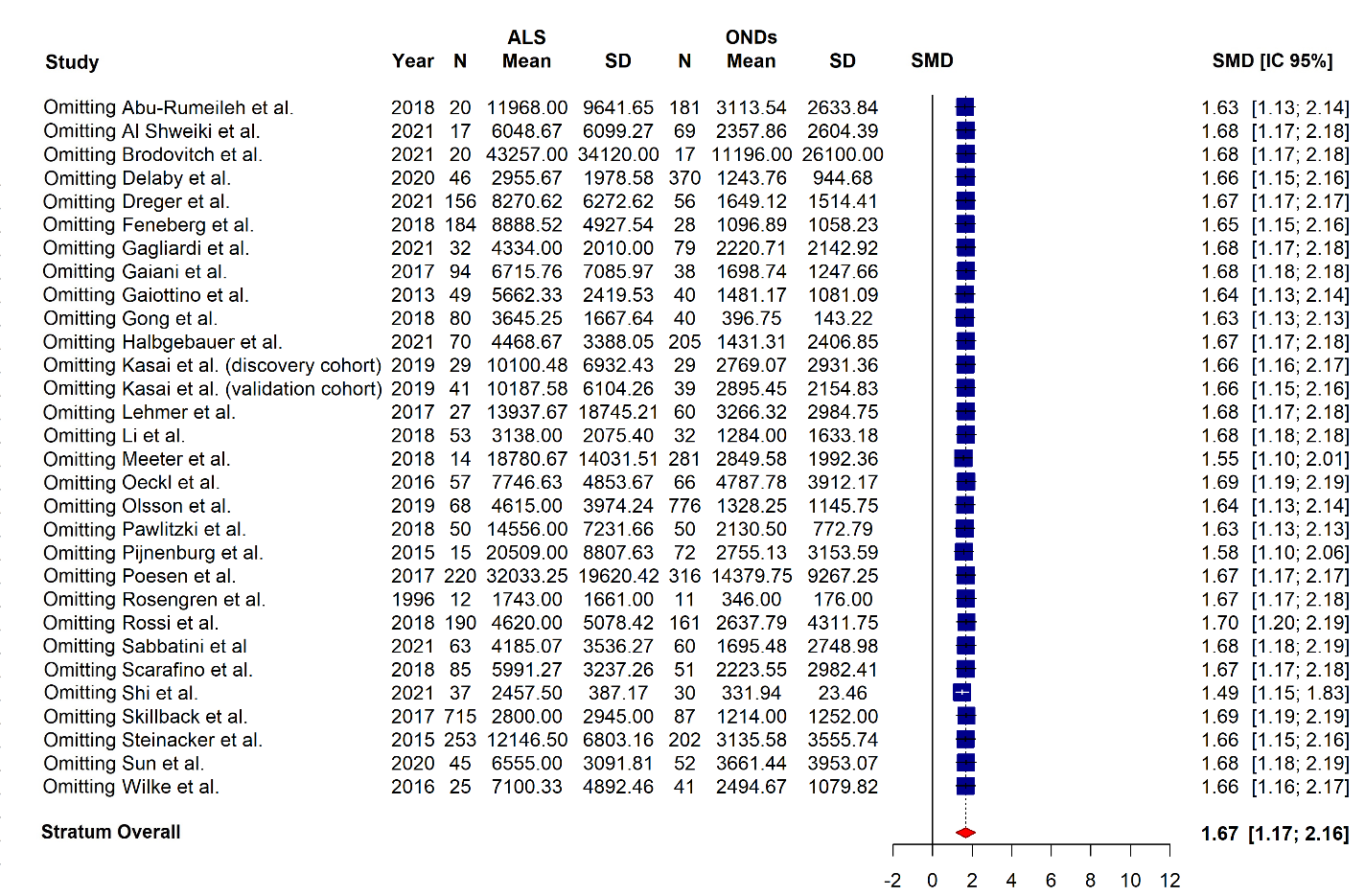 |
| Panel B: Blood |
| 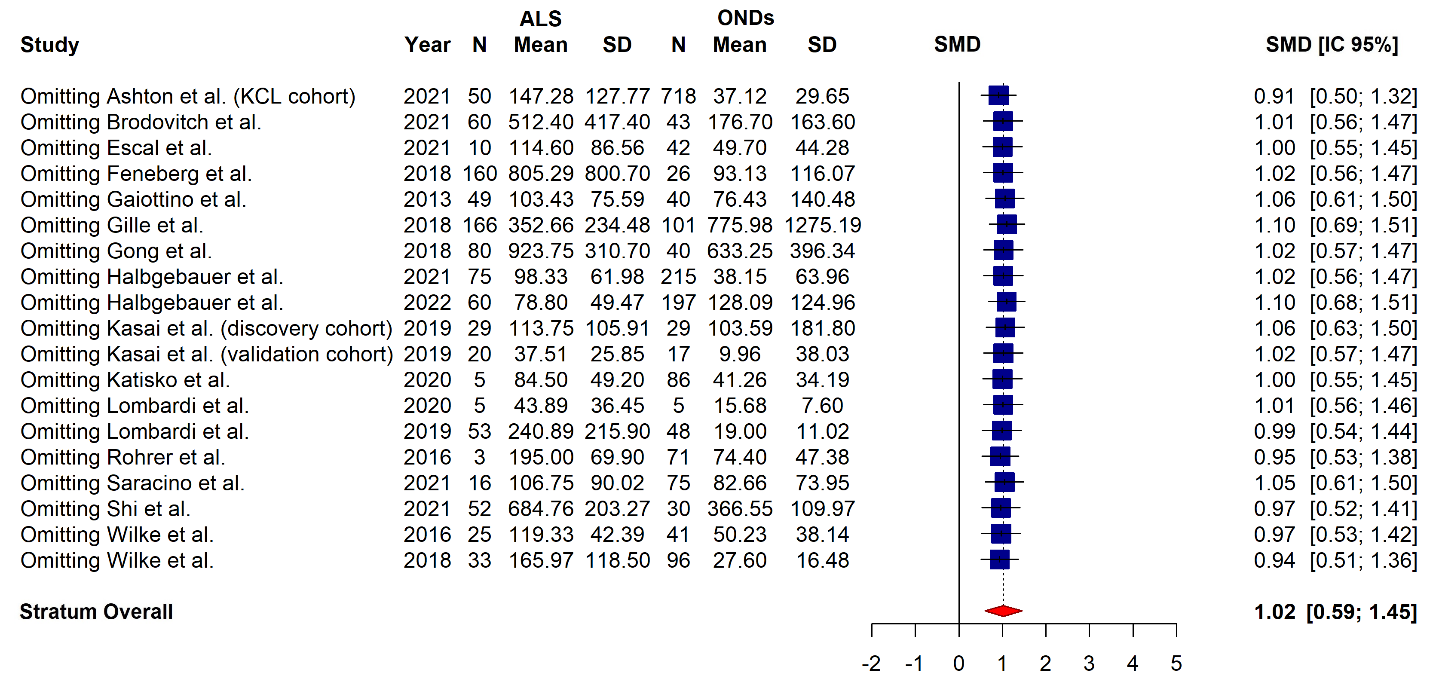 |

**Figure S18**. Influence analysis for the comparison of CSF and blood NFL levels in bvFTD vs. NHCs.

| Panel A: CSF |
| --- |
| 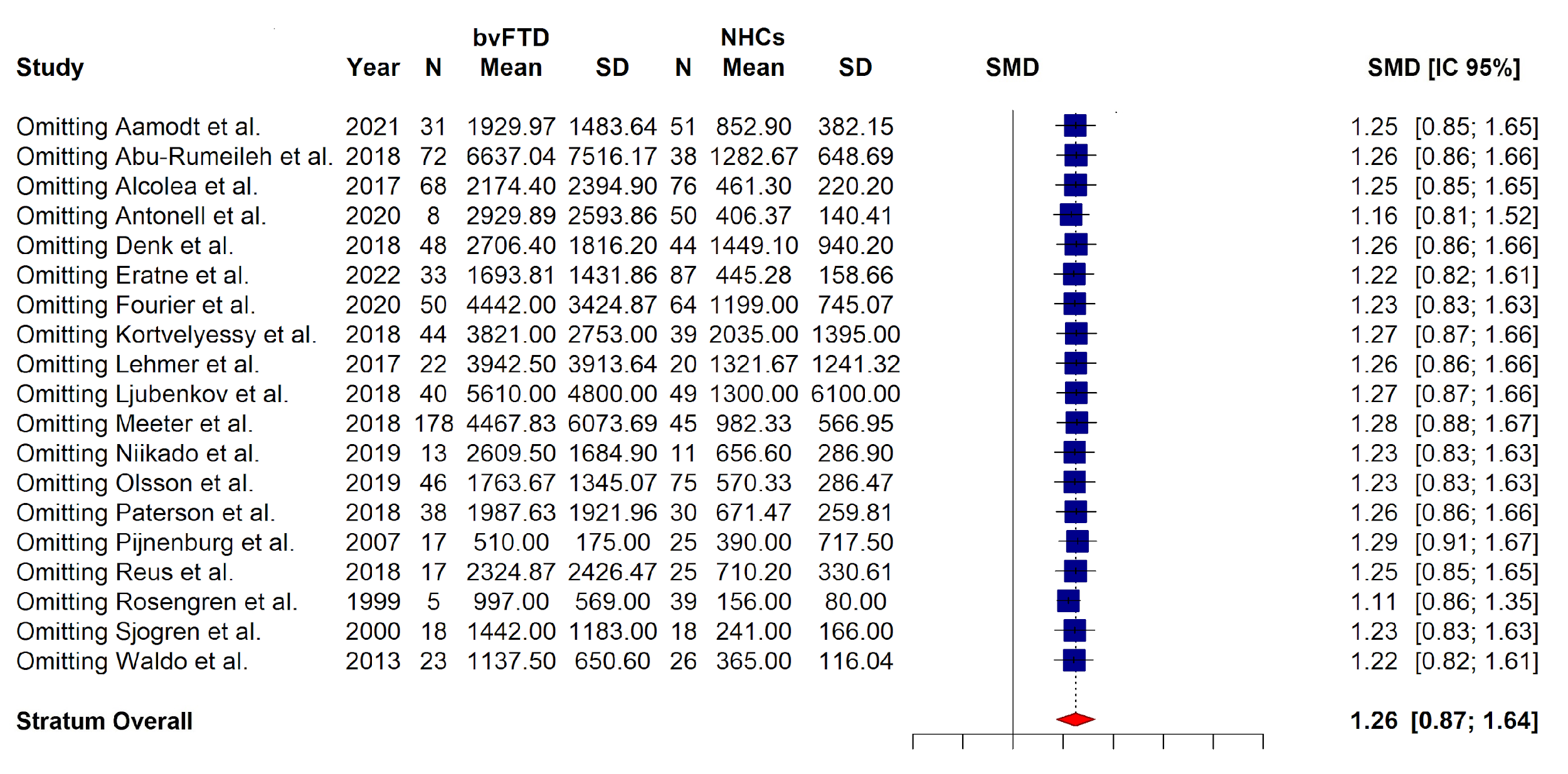 |
| Panel B: Blood |
| 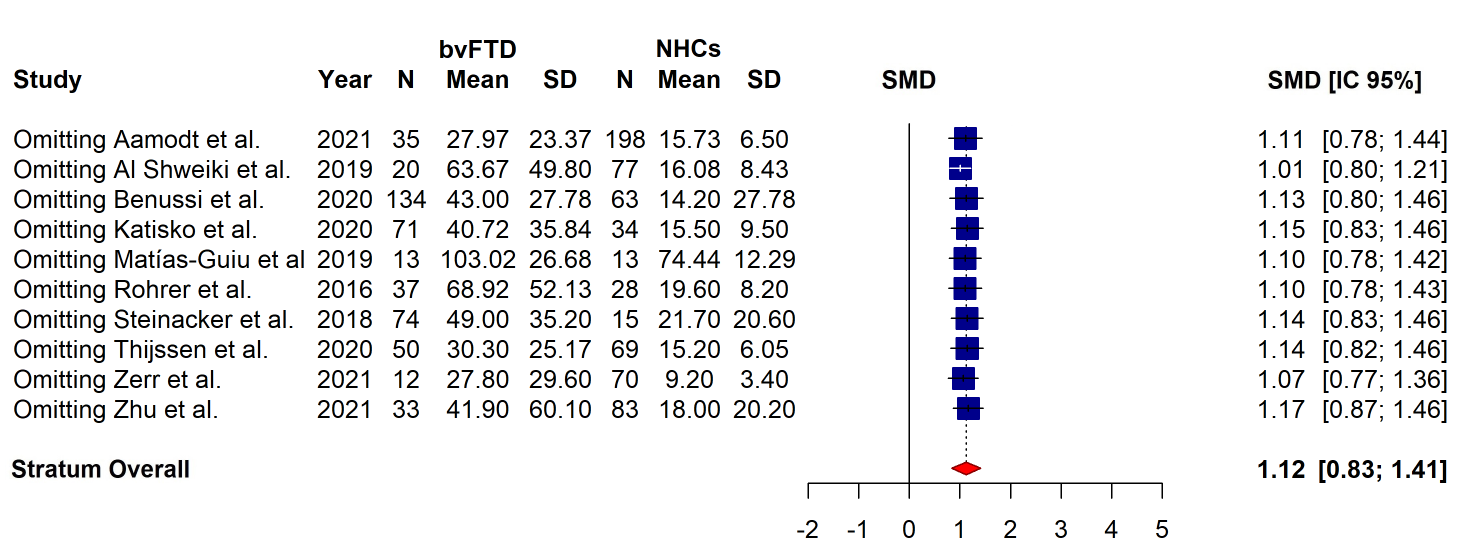 |

**Figure S19**. Influence analysis for the comparison of CSF and blood NFL levels in bvFTD vs. ODs.

| Panel A: CSF |
| --- |
| 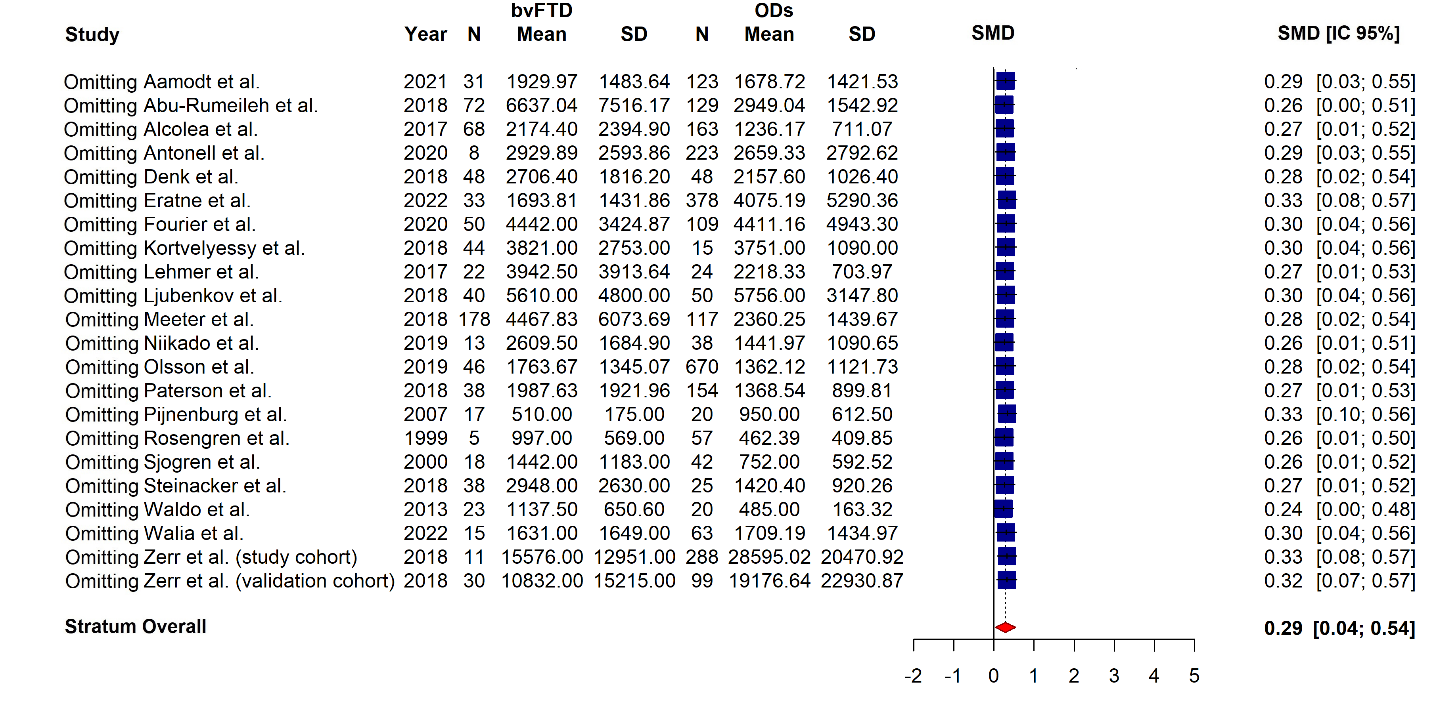 |
| Panel B: Blood |
| 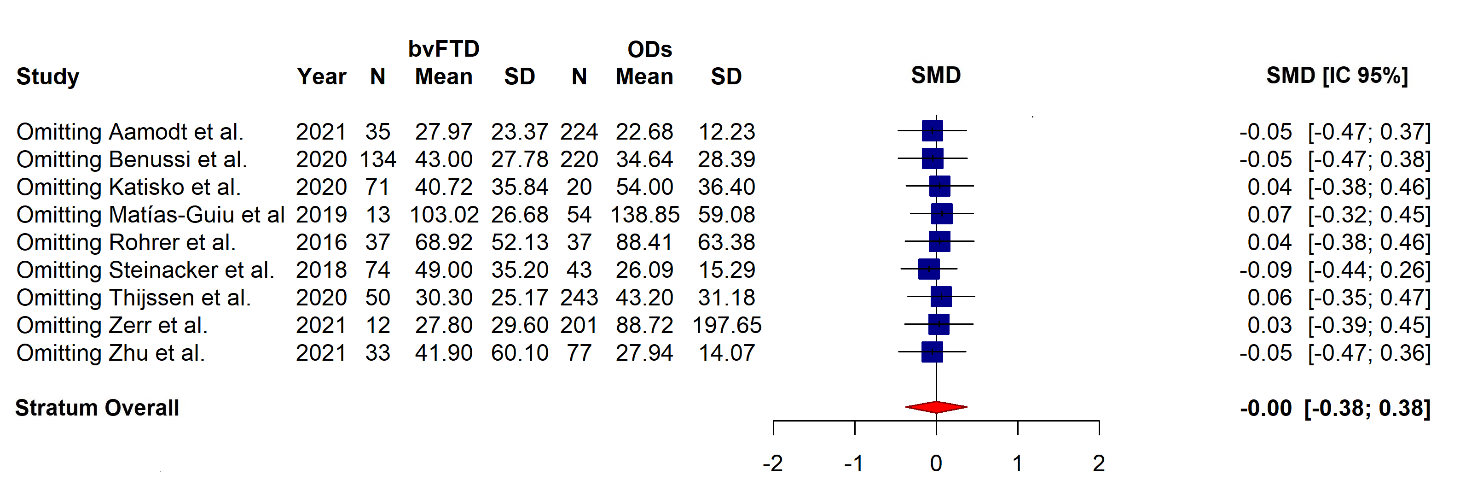 |

**Figure S20**. Influence analysis for the comparison of CSF NFL levels in bvFTD vs. ONDs.

| 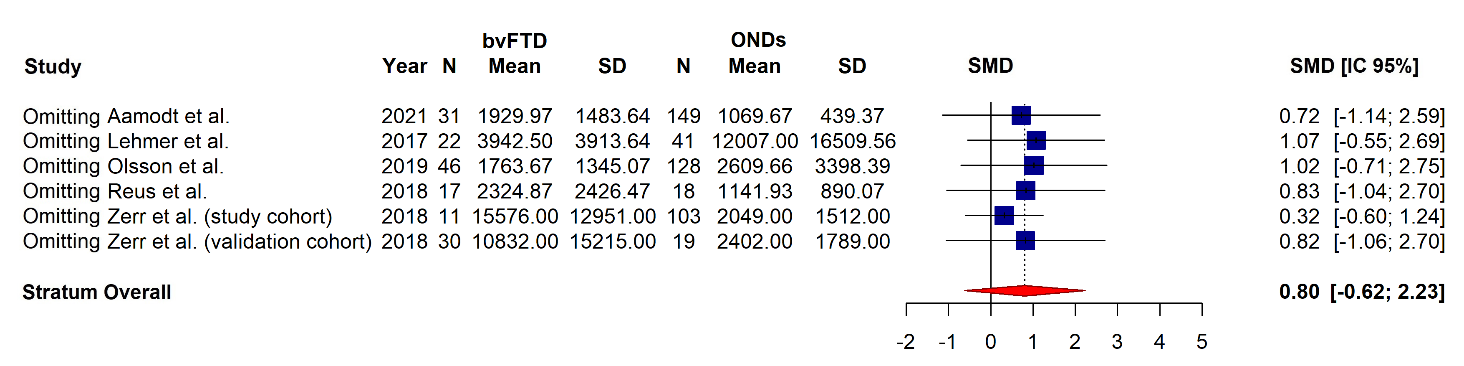 |
| --- |

**Figure S21**. Influence analysis for the comparison of CSF and blood NFL levels in FTD vs. NHCs.

| Panel A: CSF |
| --- |
| 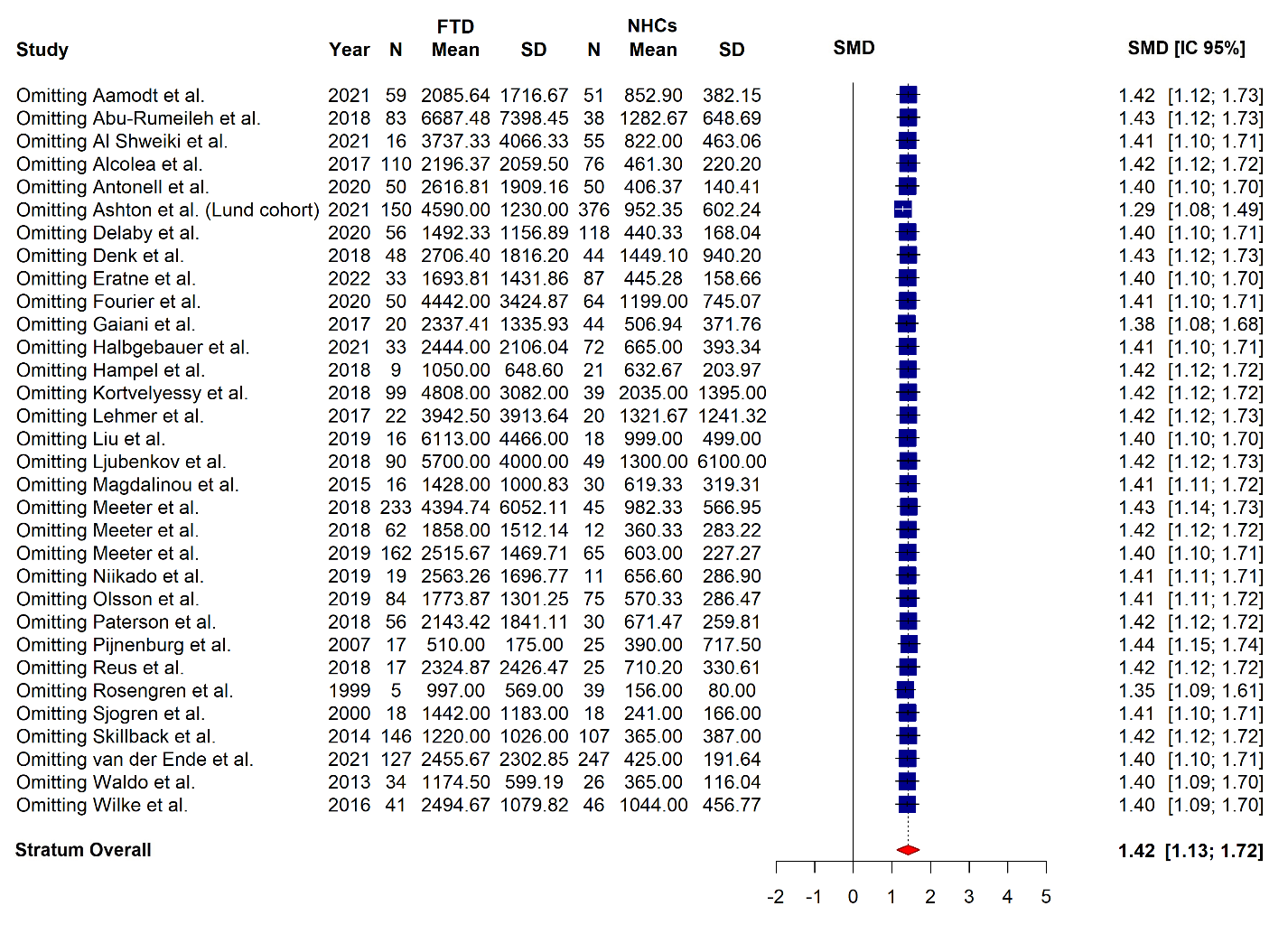 |
| Panel B: Blood |
| 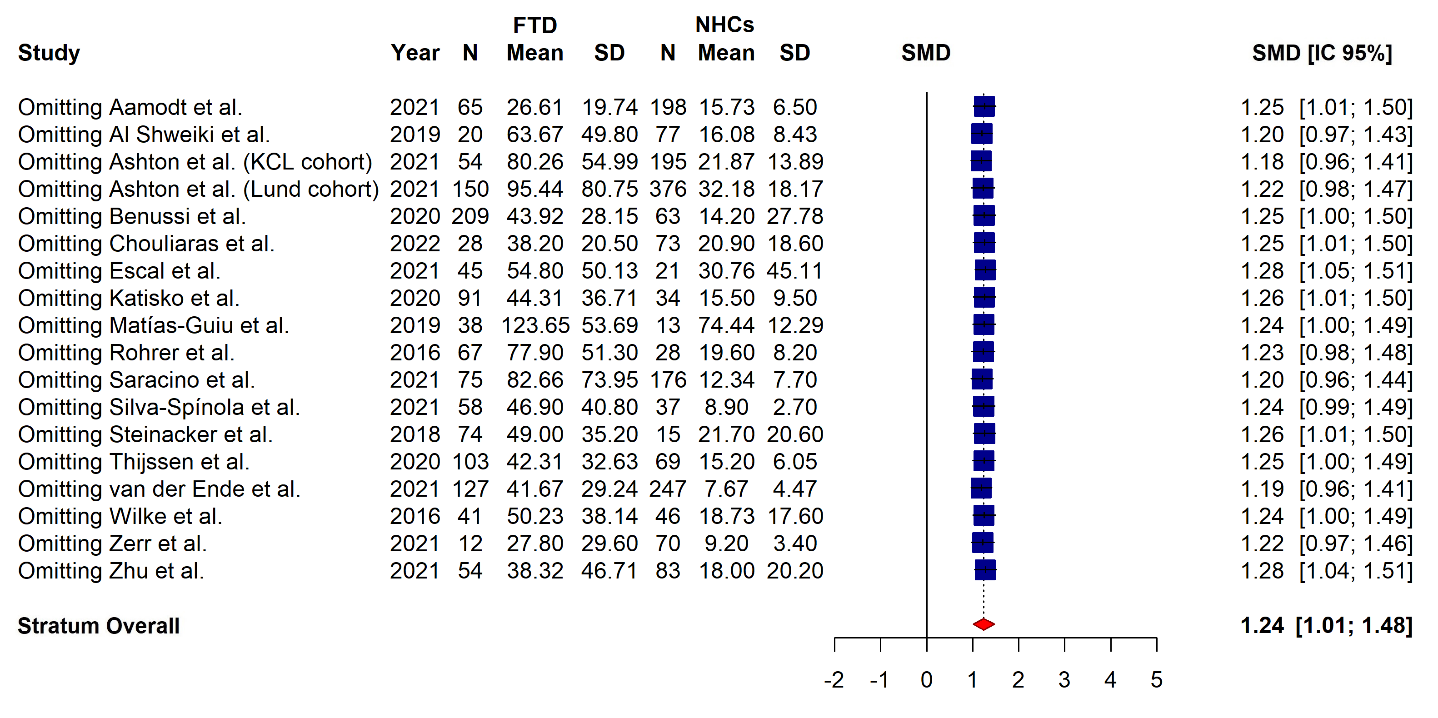 |

**Figure S22**. Influence analysis for the comparison of CSF and blood NFL levels in FTD vs. ODs.

| Panel A: CSF |
| --- |
| 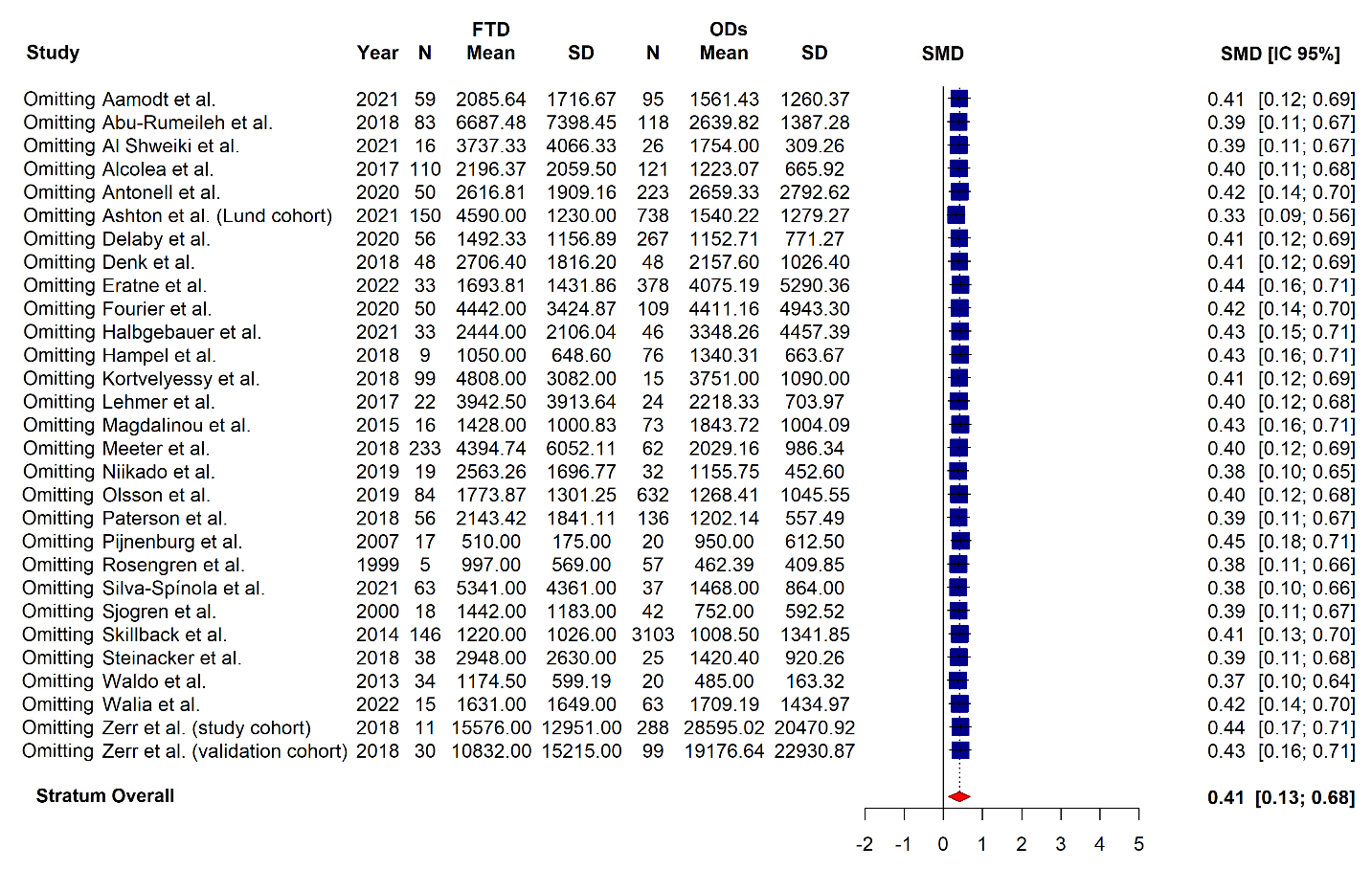 |
| Panel B: Blood |
| 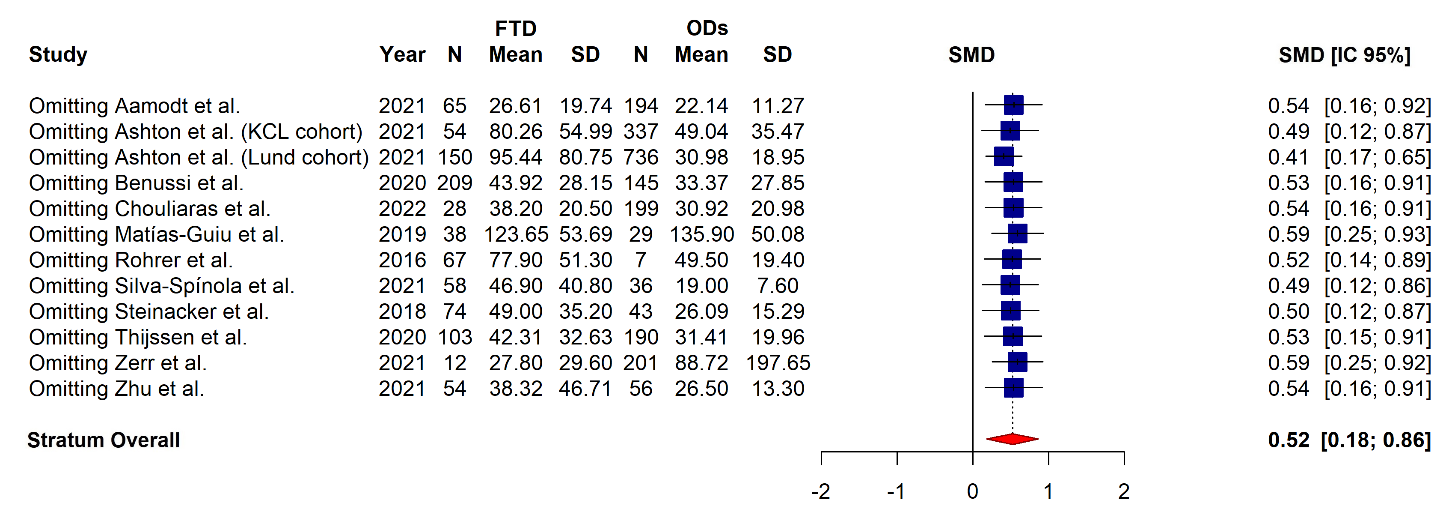 |

**Figure S23**. Influence analysis for the comparison of CSF and blood NFL levels in FTD vs. ONDs.

| Panel A: CSF |
| --- |
| 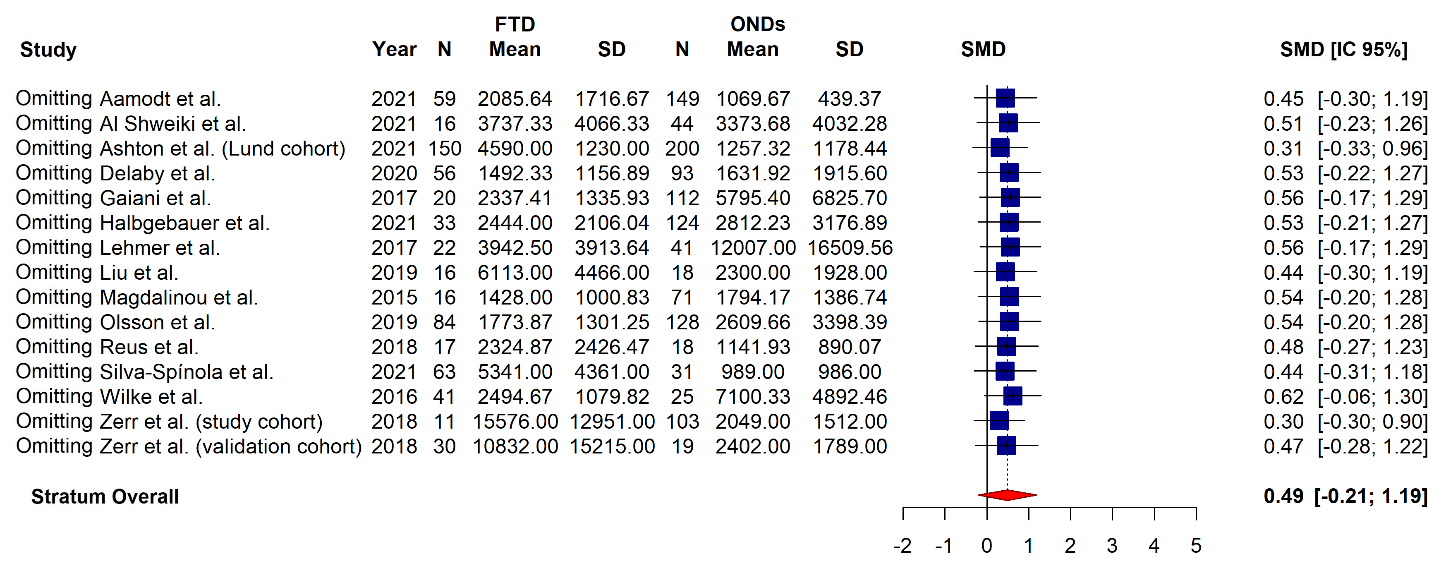 |
| Panel B: Blood |
| 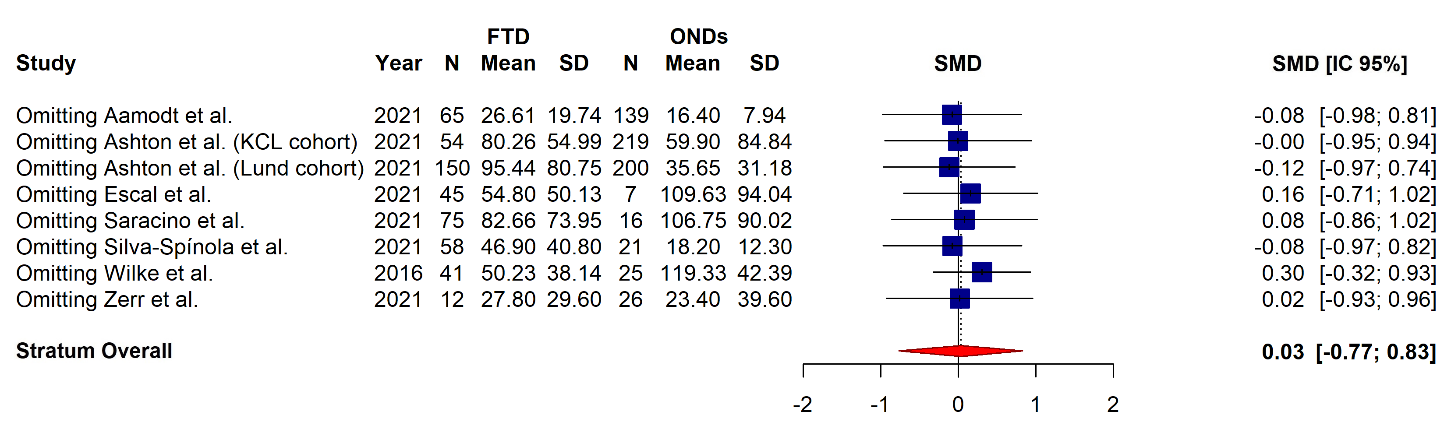 |

**Figure S24**. Influence analysis for the comparison CSF and blood NFL levels in FTLDSs vs. NHCs.

| Panel A: CSF |
| --- |
| 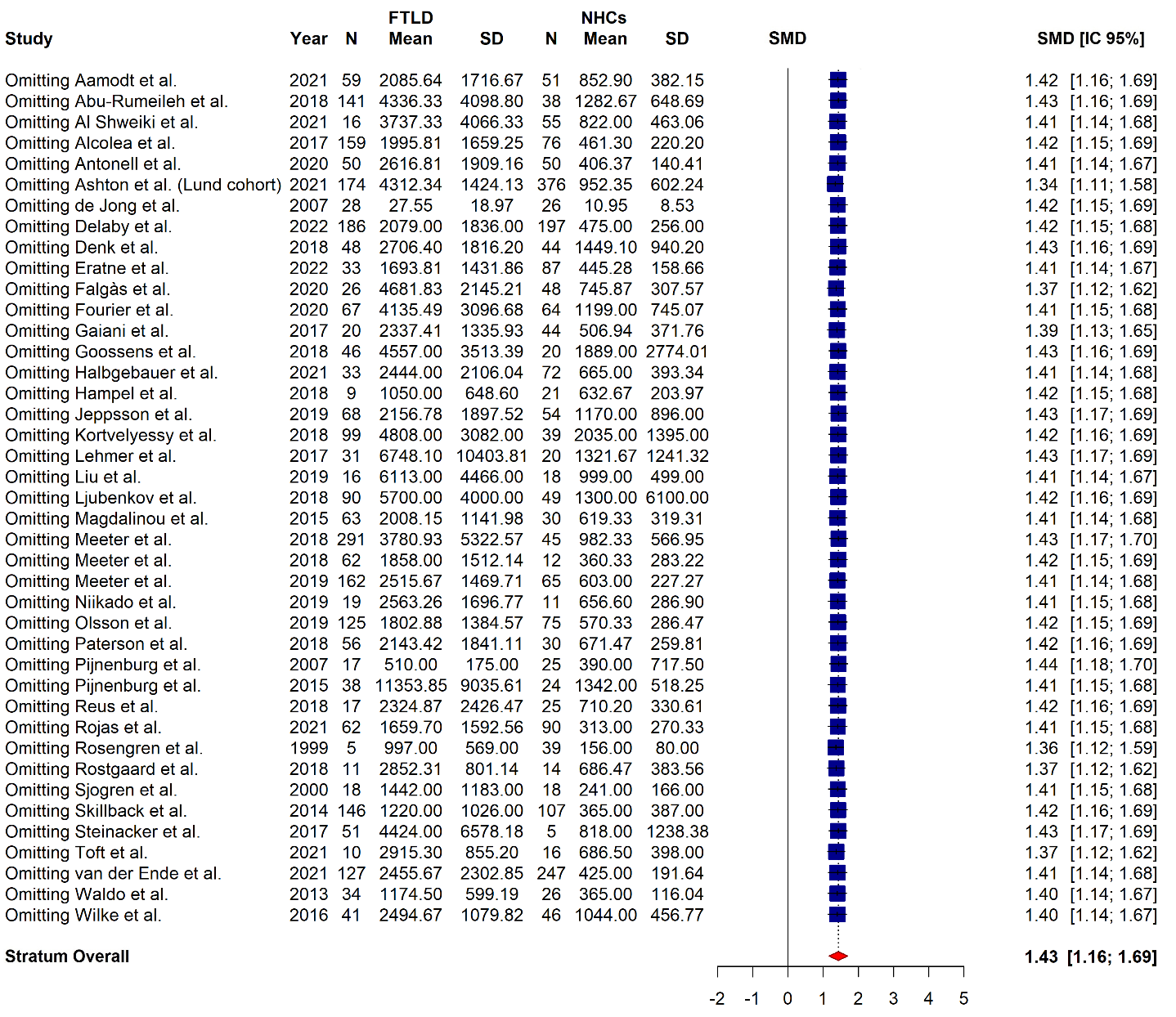 |
| Panel B: Blood |
| 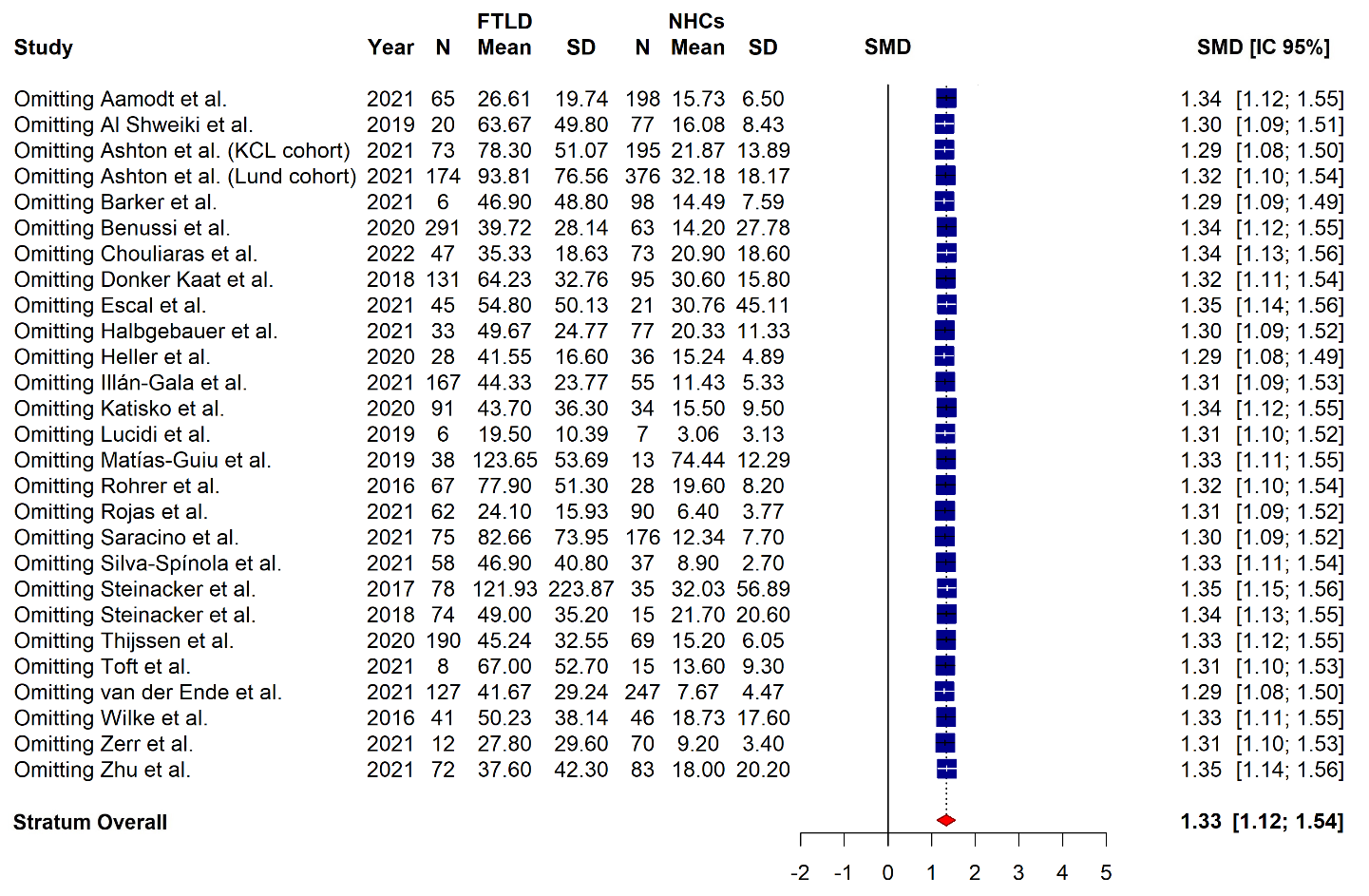 |

**Figure S25**. Influence analysis for the comparison of CSF and blood NFL levels in FTLDSs vs. ODs.

| Panel A: CSF |
| --- |
| 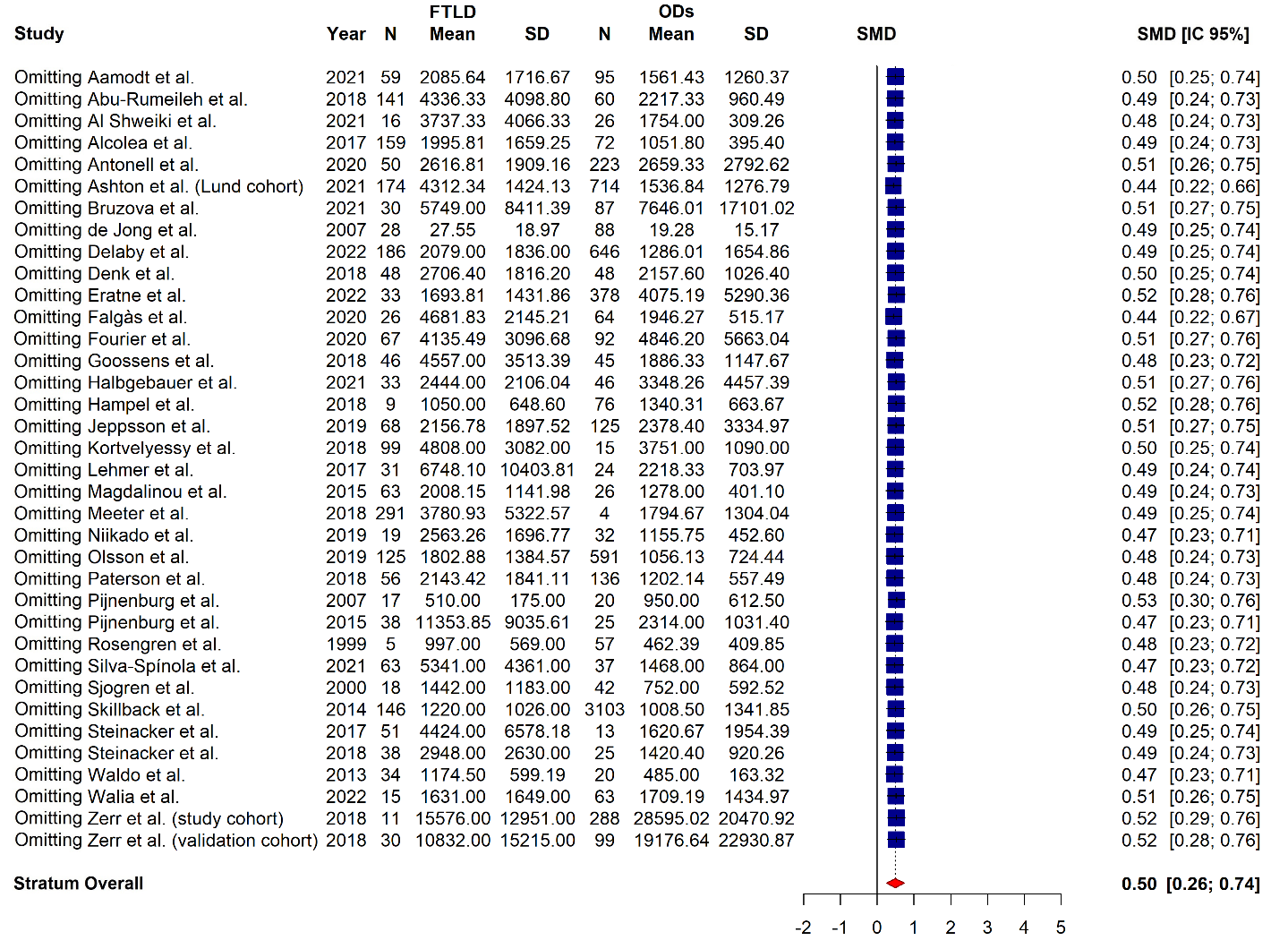 |
| Panel B: Blood |
| 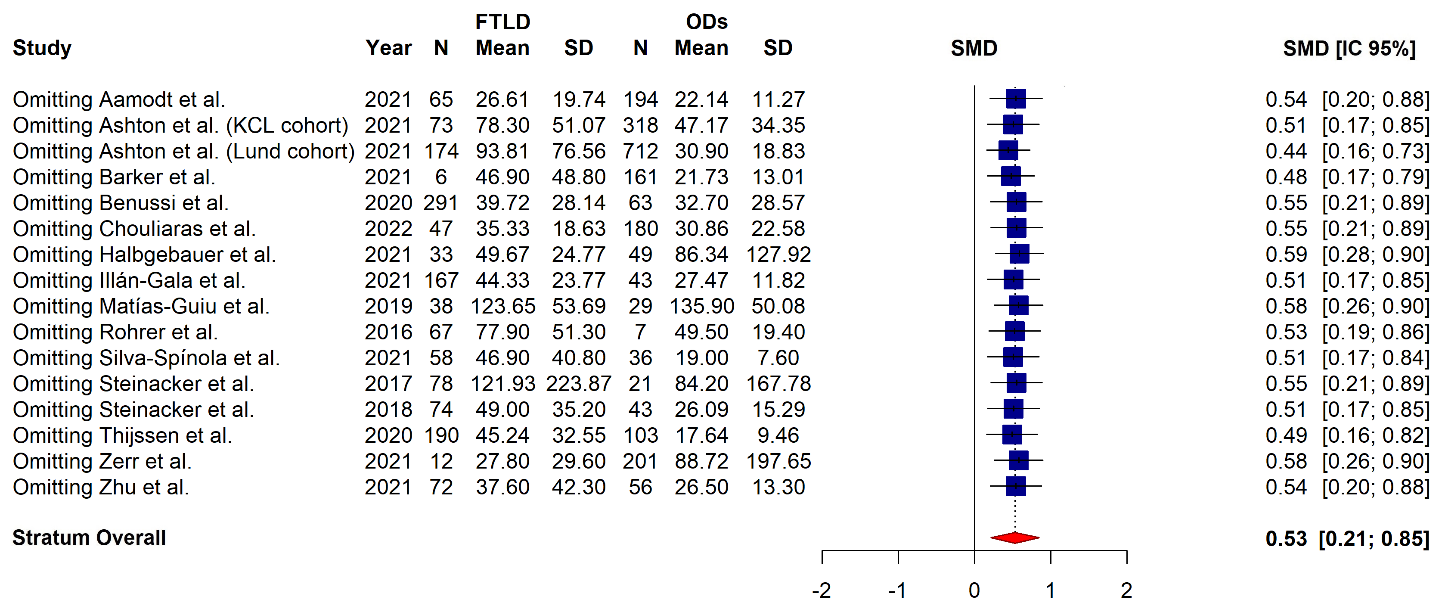 |

**Figure S26**. Influence analysis for the comparison of CSF and blood NFL levels in FTLDSs vs. ONDs.

| Panel A: CSF |
| --- |
| 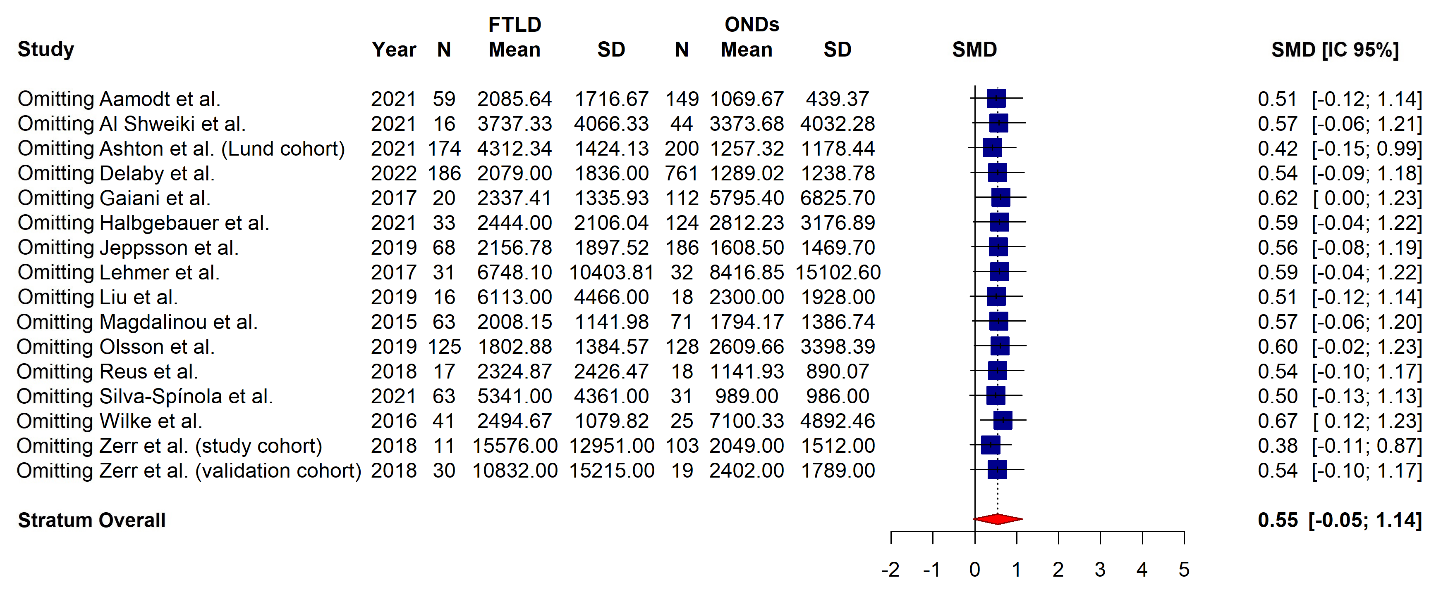 |
| Panel B: Blood |
| 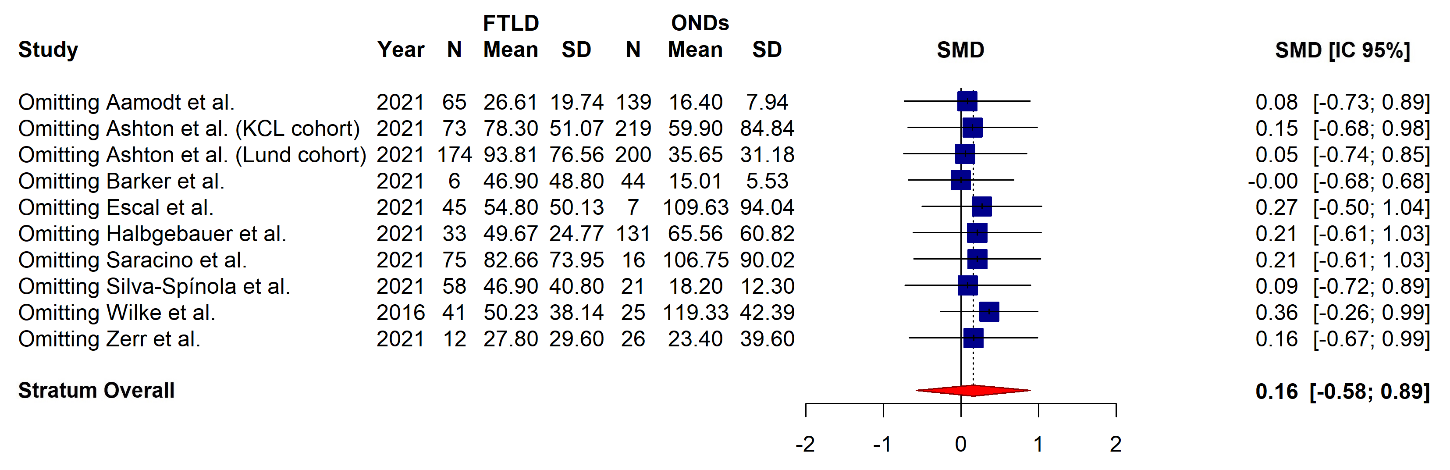 |

**Figure S27**. Funnel plots and p-values of Egger’s tests.

|  | **ALS vs NHCs** | **ALS vs AMs** | **ALS vs ONDs** | **bvFTD vs NHCs** | **bvFTD vs ODs** | **bvFTD vs ONDs** |
| --- | --- | --- | --- | --- | --- | --- |
| CSF | 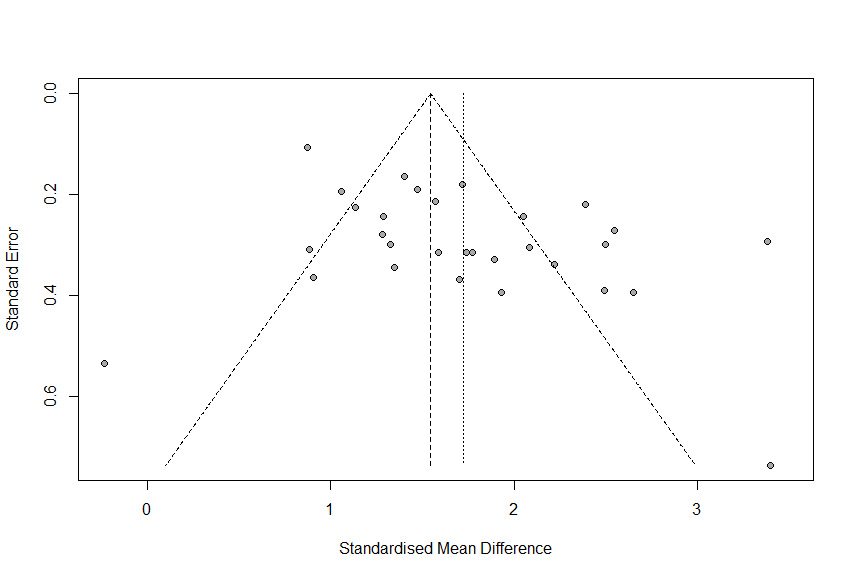 | 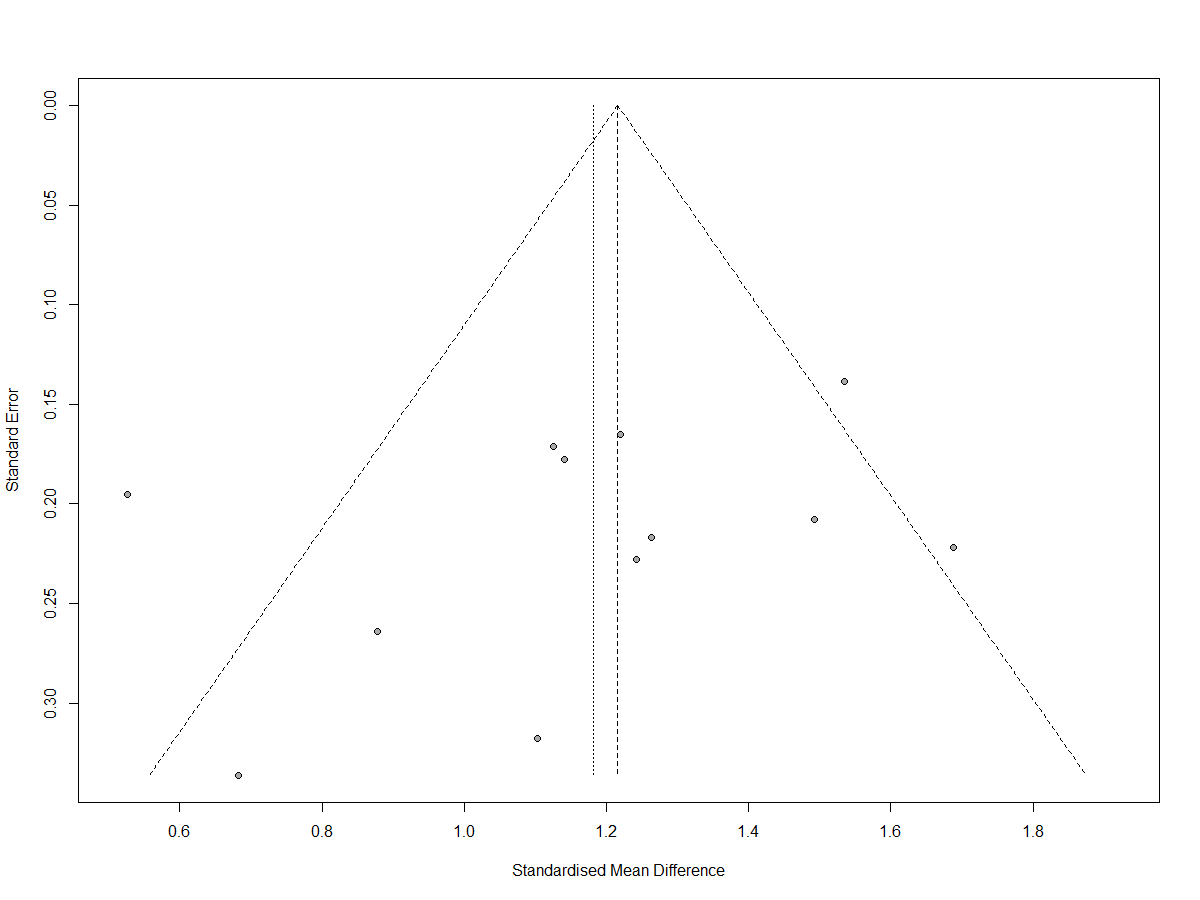 | 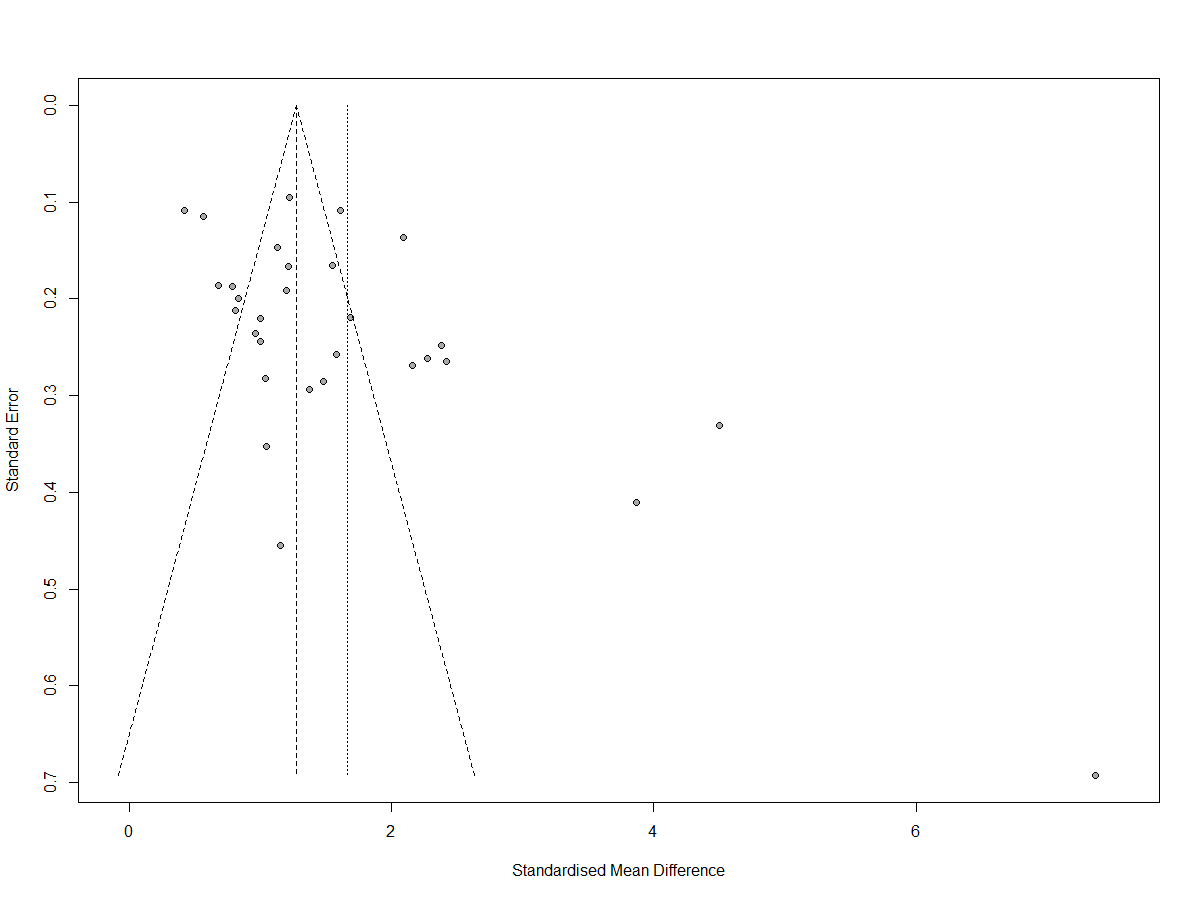 | 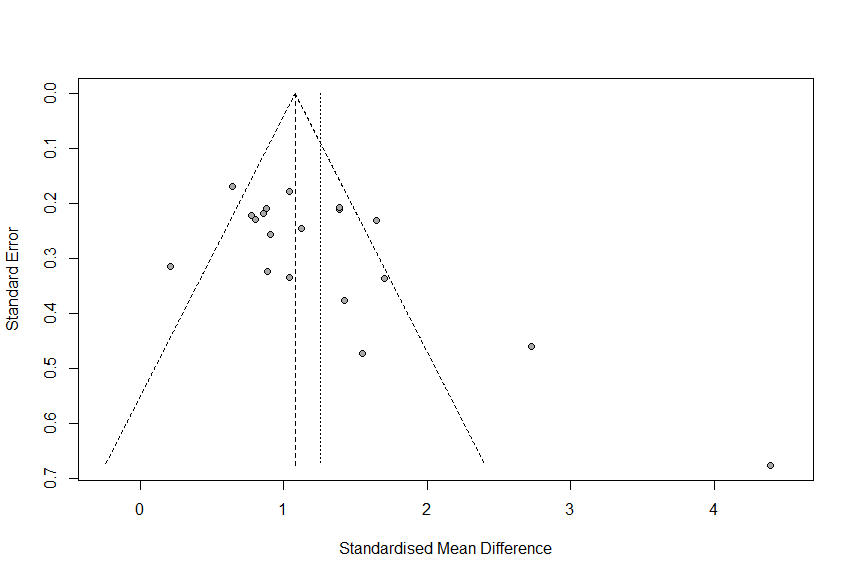 | 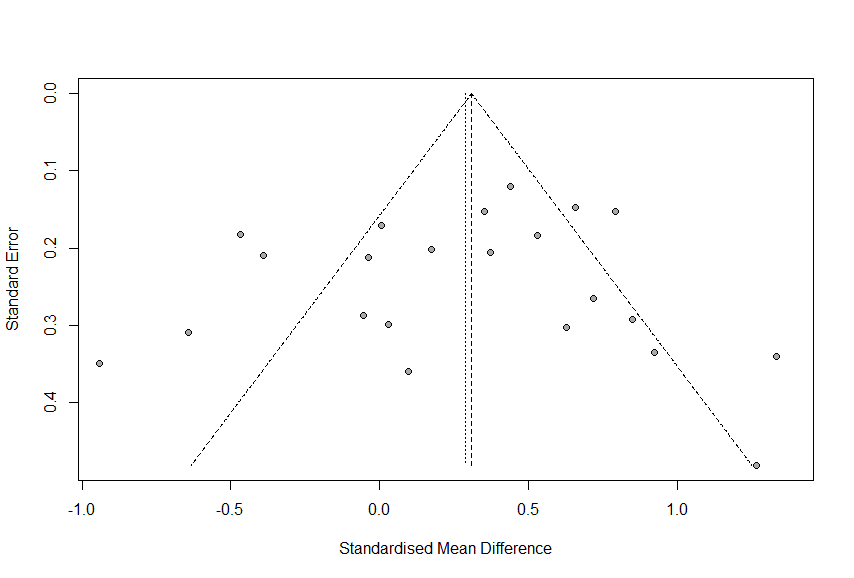 | NA |
| p-value | 0.0077 | 0.2487 | 0.0054 | 0.0102 | 0.7019 |  |
| Blood | 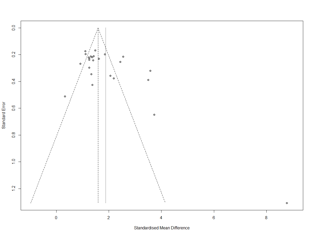 | NA | 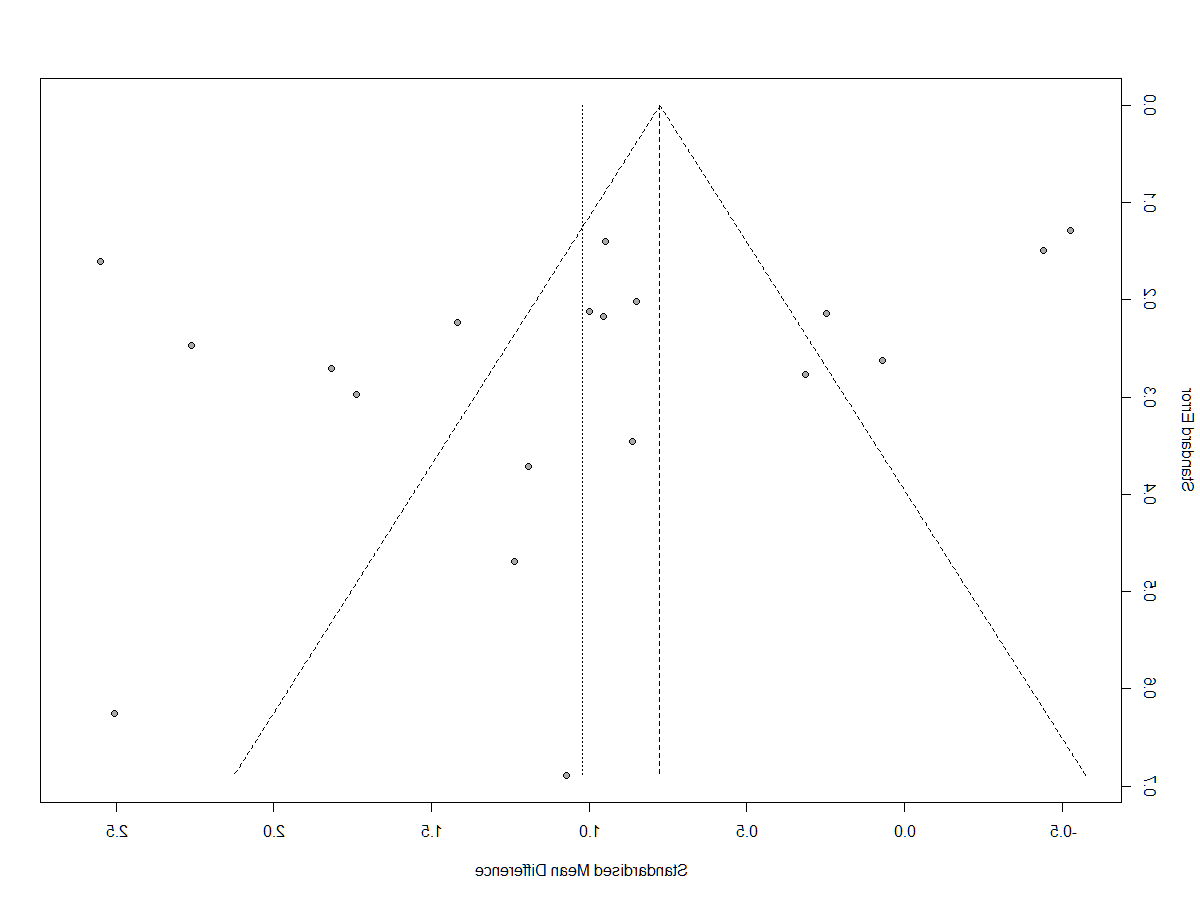 | 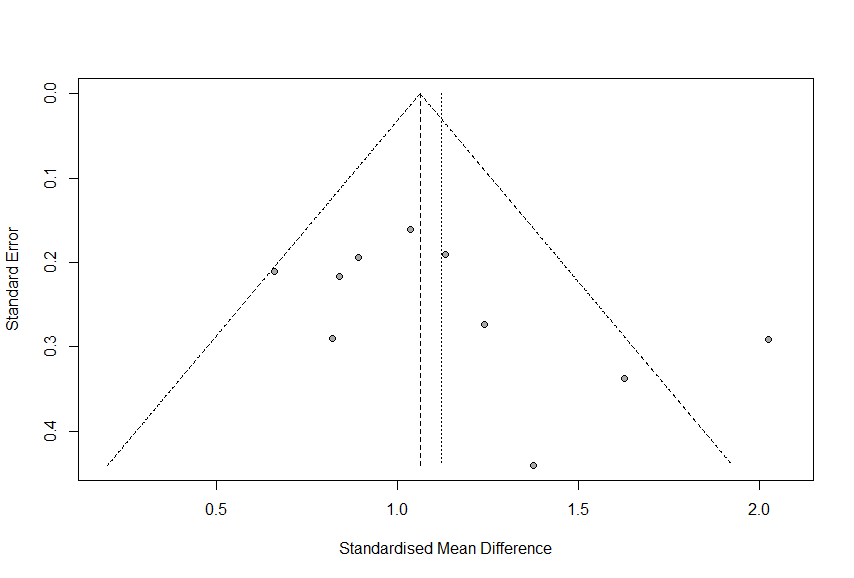 | NA | NA |
| p-value | 0.0139 |  | 0.1621 | 0.1477 |  |  |
|  | **FTD vs NHCs** | **FTD vs ODs** | **FTD vs ONDs** | **FTLDSs vs NHCs** | **FTLDSs vs ODs** | **FTLDSs vs ONDs** |
| CSF | 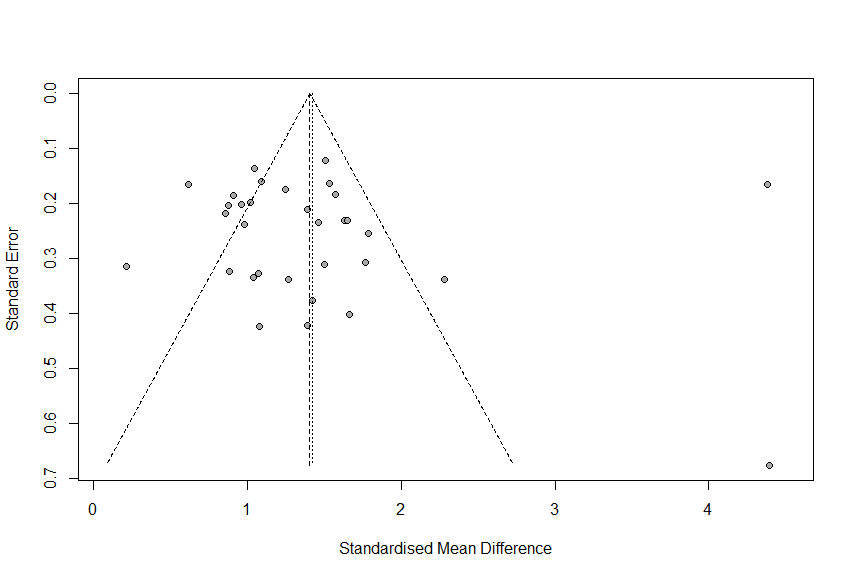 | 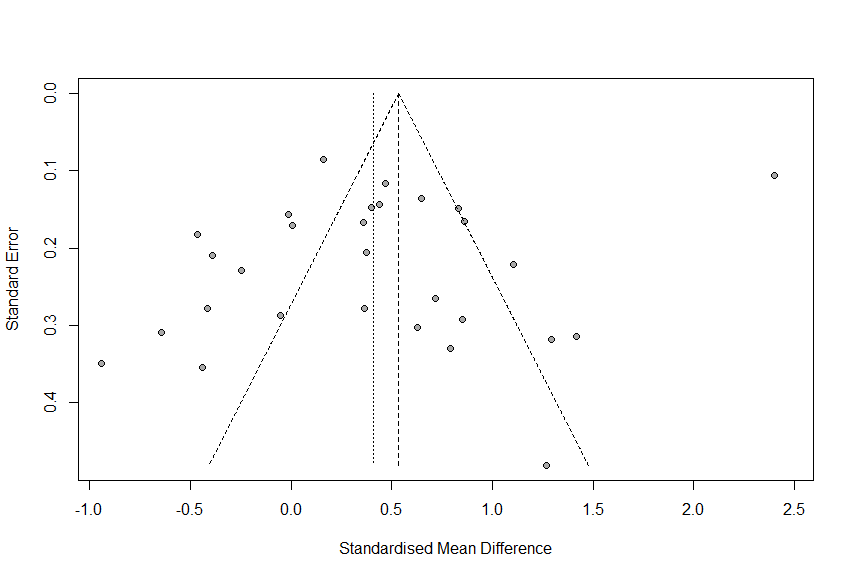 | 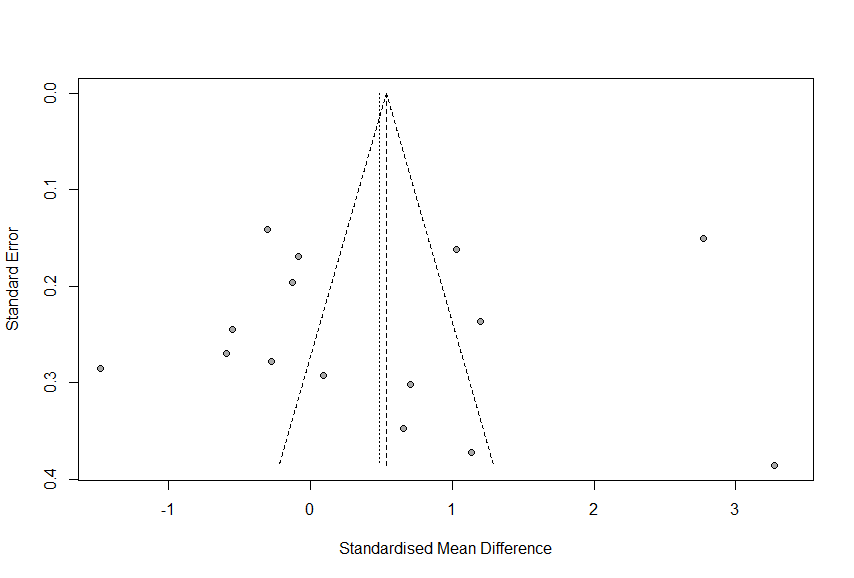 | 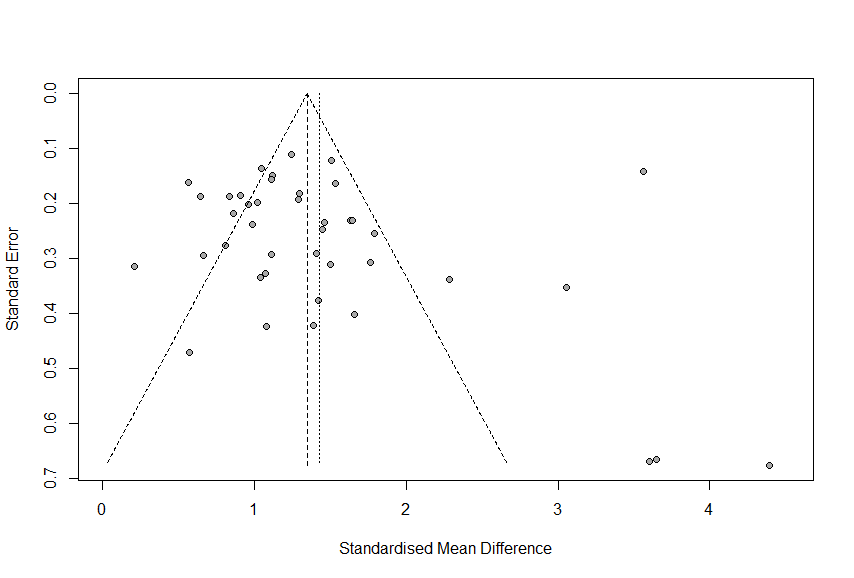 | 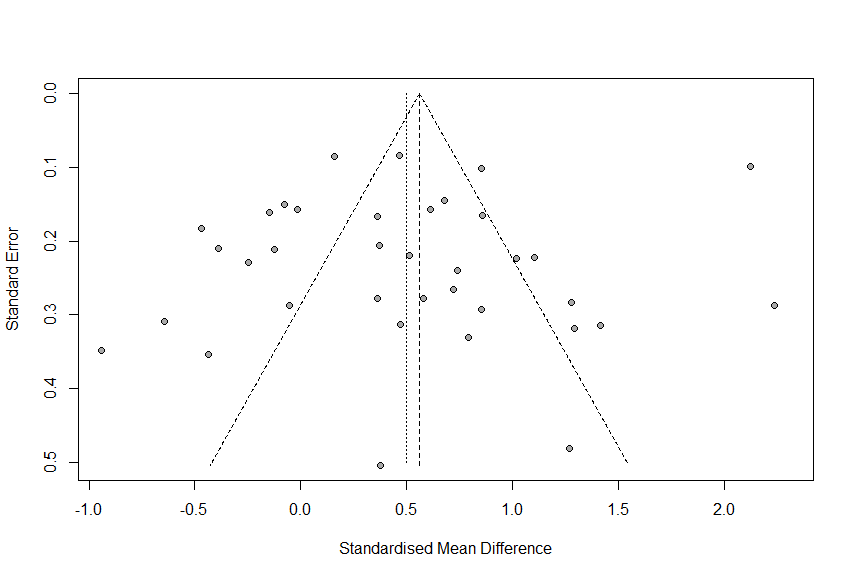 | 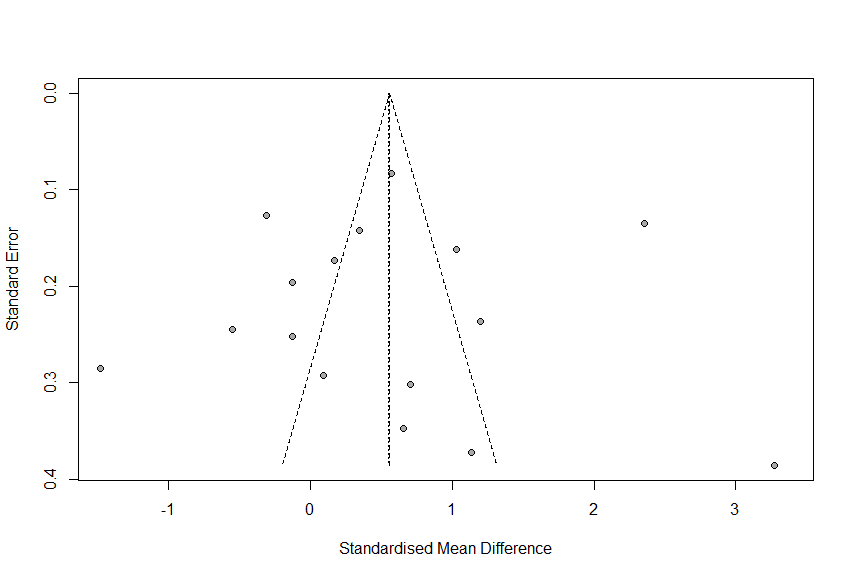 |
| P-value | 0.9574 | 0.2564 | 0.7064 | 0.5473 | 0.4624 | 0.8635 |
| Blood | 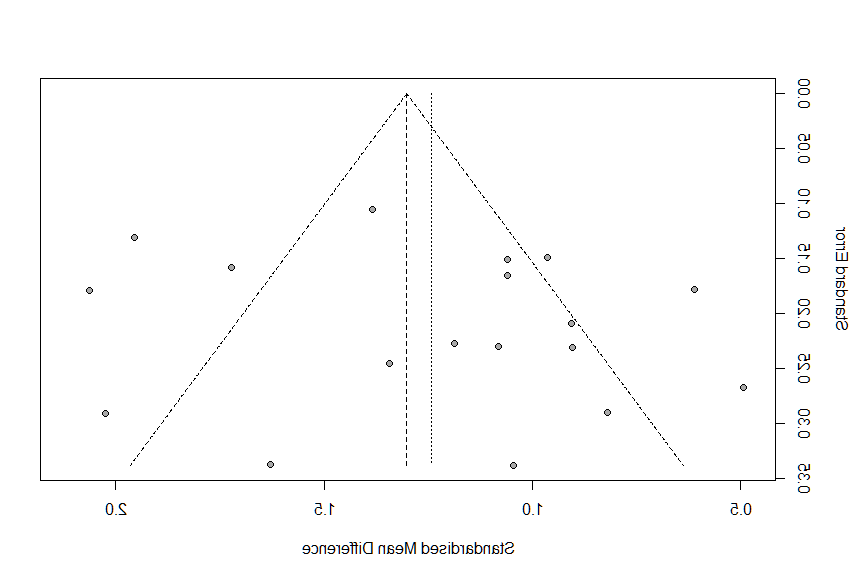 | 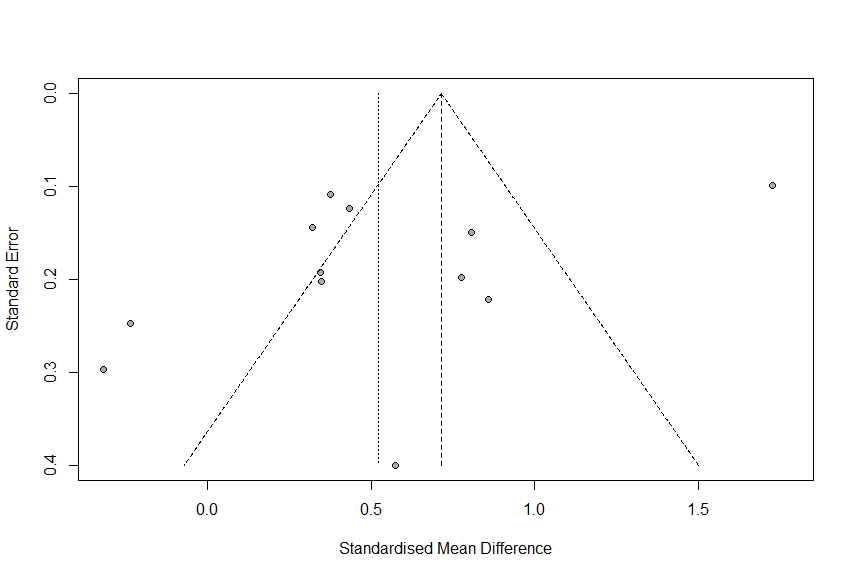 | NA | 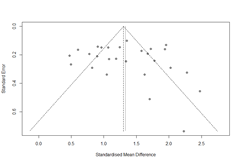 | 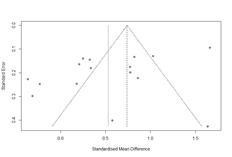 | 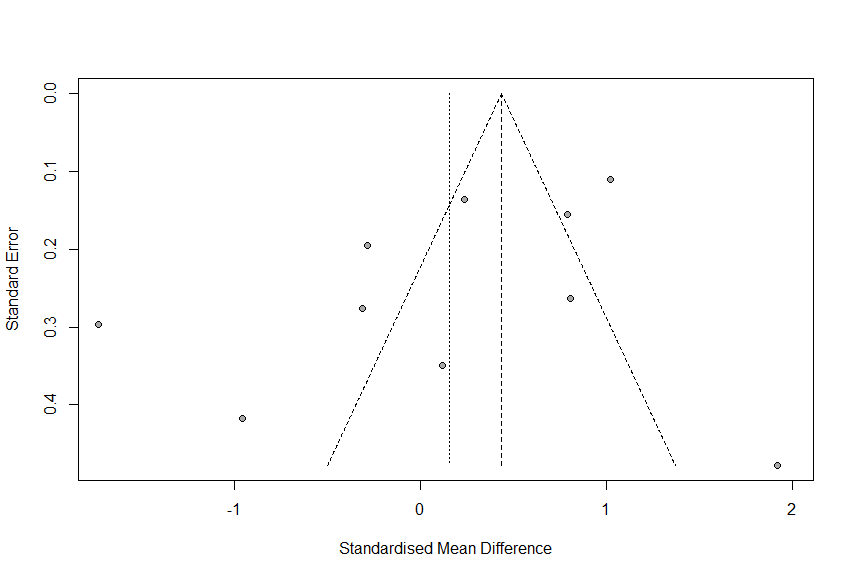 |
| p-value | 0.3212 | 0.1095 |  | 0.6868 | 0.0282 | 0.1769 |

NA: not applicable.

**Table S1**. Results of comparison-specific meta-regression model.

| **Comparisons** | **CSF** | **Blood** |
| --- | --- | --- |
| ALS vs. NHCs | None | None |
| ALS vs. AMs | NA | NA |
| ALS vs. ONDs | None | None |
| bvFTD vs. NHCs | *Number of cases*  β = -0.0076; p-value = 0.0240  *Proportion of male in cases*  β = -0.0491; p-value = 0.0213 | NA |
| bvFTD vs. ODs | None | NA |
| bvFTD vs. ONDs | NA | NA |
| FTD vs. NHCs | *Number of controls*  β = 0.0078; p-value < 0.001  *Proportion of male in cases*  β = -0.0211; p-value = 0.0507 | None |
| FTD vs. ODs | None | *Number of controls*  β = 0.0029; p-value = 0.0138 |
| FTD vs. ONDs | None | NA |
| FTLDSs vs. NHCs | *Number of controls*  β = 0.0056; p-value = 0.038  *Proportion of male in cases*  β = -0.0256; p-value = 0.020 | None |
| FTLDSs vs. ODs | None | None |
| FTLDSs vs. ONDs | None | NA |

NA: not applicable. None: no statistically significant variable from meta-regression model.
